# Supplementary material for: Content of Health-Promoting Fatty Acids in Commercial Sheep, Cow and Goat Cheeses
Source: Foods. 2022 Apr 13;11(8):1116. doi: 10.3390/foods11081116 (PMC9028068; doi:10.3390/foods11081116)
Supplement: Supplementary file 1 [file foods-11-01116-s001.zip › foods-1645403-supplementary.pdf]

**Table S1** supplementary materials. Fatty acid contend in studied cheese [mg $100g^{-1}$ of fat content]	S1: Camembert l'aromatique		S2: Camembert de Caractere. Roi du Chateau		S3: BucheFondante		S4: Gorgonzola Cremoso		S5: Gorgonzola Intense		S6: Cow mountain cheese non smoked		S7: Cow mountain cheese smoked		S8: Sheep mountain cheese “oscypek”non smoked		S9: Sheep mountain cheese “oscypek” smoked		S10: L'amidu Chambertin	
	x	SD	X	SD	X	SD	X	SD	X	SD	X	SD	X	SD	X	SD	X	SD	X	SD
C4:0	3442.40	131.83	3938.25	286.55	4624.83	381.28	2230.92	116.49	3652.92	485.12	4202.16	210.39	4438.90	594.31	3601.79	311.30	4576.09	593.83	3291.48	395.22
C6:0	2225.58	94.67	2301.13	40.60	2481.61	93.63	1869.59	34.49	2177.25	46.03	2096.78	9.38	2106.49	13.02	2305.24	166.95	2518.76	29.74	2111.26	127.47
C8:0	1628.66	50.79	1684.29	25.96	3412.83	145.19	1424.01	13.43	1626.98	38.68	1437.92	23.47	1467.12	22.32	2349.98	209.48	2644.89	30.97	1554.36	91.20
C10:0	2753.81	74.50	2760.07	45.68	8569.15	383.74	2586.71	25.96	2787.40	70.53	2151.00	42.82	2257.37	47.76	4722.28	470.82	5338.32	127.70	2893.13	179.53
C12:0	2895.85	92.69	2900.15	38.27	3735.88	180.49	2862.05	28.15	2973.22	59.84	2181.00	31.51	2323.88	23.57	2856.88	178.40	3085.32	52.45	3164.52	219.68
C13:0_iso	46.87	2.45	40.61	4.95	29.85	2.61	28.92	1.55	31.18	1.03	49.52	1.79	49.77	3.51	53.11	13.11	43.49	3.70	37.18	3.74
C10:1	79.49	5.85	84.87	2.20	49.08	2.60	75.43	3.66	81.13	2.13	50.40	2.12	44.46	25.71	54.70	4.91	56.57	8.43	82.40	4.87
C13:0_ai	75.61	4.98	83.80	5.43	82.56	2.63	82.37	12.63	80.58	1.59	51.27	1.71	56.37	5.56	61.51	8.88	64.54	4.99	83.12	3.25
C14:0_iso	112.62	3.22	103.54	1.94	84.46	4.56	76.21	1.47	82.06	0.86	147.29	1.22	119.65	0.14	142.94	12.98	134.32	1.79	112.86	8.02
C14:0	9743.93	332.81	9351.86	91.91	8752.35	420.51	9376.07	108.73	9417.40	158.48	8144.79	93.17	8140.02	98.85	8441.23	562.93	8418.82	72.45	10133.44	708.78
C15:O_iso	261.76	11.84	224.00	2.54	191.77	13.11	177.49	1.78	182.97	5.02	337.56	5.49	340.91	5.87	360.39	35.20	332.88	11.39	235.18	15.38
C15:O_ai	489.87	13.88	460.17	3.45	298.01	13.01	357.30	7.15	381.74	7.63	523.03	4.96	488.04	7.27	648.67	53.71	626.38	2.01	518.29	34.46
C14:1_c9_(n-5)	738.22	26.99	822.26	7.24	141.97	10.10	741.03	11.46	813.65	14.02	592.33	7.29	617.10	12.48	287.71	21.42	283.24	4.17	692.66	50.79
C15:0	1010.04	28.96	1009.15	5.95	829.44	54.75	1013.27	12.49	985.63	11.33	1106.66	14.55	1001.86	24.71	1163.53	85.82	1136.40	14.92	1072.58	83.80
C16:0_iso	37.70	3.46	37.82	6.33	53.67	6.89	85.19	103.87	28.41	2.10	58.57	1.22	68.85	1.50	77.12	7.49	67.46	1.83	41.58	2.88
C16:0_ai	309.57	11.18	263.58	10.85	231.64	24.08	223.08	25.53	220.20	2.02	312.30	8.46	273.78	1.16	292.96	25.81	278.10	0.87	275.71	10.96
C15:1_n10_(n-5)	16.42	0.67	14.80	9.52	14.00	1.75	20.11	6.06	19.44	5.65	17.06	1.50	17.16	1.65	15.87	0.68	17.35	0.70	15.57	2.28
C16:0	24272.84	845.28	25509.78	216.30	22218.53	1129.87	26475.35	350.77	25991.43	422.51	23535.32	268.78	21989.84	273.67	19385.58	1484.38	18798.42	27.58	24465.18	1683.70
C17:O_iso	438.51	17.63	371.58	4.41	391.26	18.89	320.15	4.67	322.57	9.19	516.49	8.70	504.82	9.00	764.25	40.17	762.55	2.16	385.50	25.94
C17:O_ai	212.01	10.10	204.05	5.60	233.10	10.65	127.88	0.88	145.38	5.46	244.82	5.81	253.57	6.59	334.02	29.38	307.87	6.15	157.48	10.91
C16:1_t9	414.99	11.61	404.88	0.71	339.57	11.27	345.11	8.99	347.15	5.59	432.65	2.21	393.05	13.28	457.20	51.10	416.57	2.01	438.33	35.21
C16:1_c9_(n-7)	1085.79	50.96	1374.02	18.88	505.23	33.29	1140.58	11.58	1287.69	27.21	1338.49	18.94	1285.33	17.46	766.02	80.54	712.14	11.67	1045.91	72.54
C16:1_t10	71.03	4.54	56.48	5.91	26.97	3.19	24.96	1.78	30.22	2.48	115.93	1.96	125.03	3.91	83.23	9.29	0.00	0.00	39.20	3.35
C16:1_t11_+_t12	48.97	16.81	52.14	4.23	15.35	1.37	32.88	1.73	31.82	9.47	42.17	1.04	36.23	1.68	39.70	39.02	75.67	0.98	30.94	7.40
C17:0	624.48	21.72	634.18	7.38	617.54	31.57	607.20	5.77	590.23	16.87	787.60	8.61	681.31	9.90	708.13	75.33	650.11	5.08	617.91	42.08
C18:0_iso	61.09	4.53	62.15	1.27	43.40	1.96	42.13	12.79	45.99	1.83	88.37	4.59	77.45	1.70	76.41	8.25	73.50	4.51	53.77	3.82
C18_0_ai	34.33	0.06	37.18	4.80	42.79	6.09	75.17	81.39	31.20	3.00	36.20	2.26	36.34	0.96	39.92	7.60	30.62	11.76	31.90	5.56
C17:1_c9	43.33	0.52	39.42	3.84	26.98	2.23	15.26	3.08	14.94	2.86	67.90	2.06	61.58	2.40	71.46	9.01	59.20	5.80	27.49	2.57
C17:1_c10_(n-7)	174.19	3.90	225.32	1.62	173.73	5.62	175.21	9.90	189.40	9.46	309.22	3.93	262.67	5.96	237.65	24.82	207.48	5.07	165.70	10.17
C18:0	10041.20	354.95	8875.28	55.34	8668.93	470.70	8790.27	105.18	8695.93	150.28	10932.73	146.20	10725.41	163.47	9868.77	787.44	9277.59	165.72	8046.00	553.62
C18:1_t6	20.35	0.92	20.91	2.07	17.63	0.19	21.32	0.49	21.13	0.94	18.02	2.30	17.36	2.79	11.99	8.41	11.56	10.42	19.93	1.46
C18:1_t7	19.61	4.75	23.19	2.59	25.78	8.02	23.07	7.61	22.41	1.69	17.09	2.12	22.07	6.06	24.17	5.47	20.84	6.14	22.10	2.72

**Table S1** supplementary materials. Fatty acid contend in studied cheese [mg 100g^{-1} of fat content]	S1: Camembert l'aromatique		S2: Camembert de Caractere. Roi du Chateau		S3: BucheFondante		S4: Gorgonzola Cremoso		S5: Gorgonzola Intense		S6: Cow mountain cheese non smoked		S7: Cow mountain cheese smoked		S8: Sheep mountain cheese “oscypek”non smoked		S9: Sheep mountain cheese “oscypek” smoked		S10: L'amidu Chambertin	
	x	SD	X	SD	X	SD	X	SD	X	SD	X	SD	X	SD	X	SD	X	SD	X	SD
C18:1_t8	203.54	10.87	180.15	12.92	217.11	11.39	237.27	10.55	240.68	0.72	167.11	6.39	154.95	2.94	230.80	8.31	228.71	1.19	213.57	14.41
C18:1_t9	221.67	9.82	207.33	6.53	262.98	16.94	236.00	4.94	255.26	4.42	196.66	2.41	190.97	9.23	288.86	9.20	292.94	14.83	224.56	19.81
C18:1_t10	0.00	0.00	208.19	24.92	348.43	17.67	576.76	18.20	575.74	21.27	0.00	0.00	0.00	0.00	0.00	0.00	0.00	0.00	0.00	0.00
C18:1_t11	2081.32	85.00	1341.03	125.47	962.33	49.36	817.75	6.67	832.22	17.08	2401.42	34.72	2324.71	42.65	4196.67	384.20	4595.67	87.46	1767.03	119.16
C18:1_t12	260.56	10.19	229.56	5.70	260.63	17.89	338.11	4.99	345.41	3.25	181.16	2.74	210.30	3.04	347.42	16.34	351.46	11.79	253.98	20.65
C18:1_t13+t14	693.53	25.21	578.75	2.34	522.40	23.90	746.37	12.39	751.68	15.63	552.16	7.78	688.48	32.13	980.88	52.04	965.41	18.70	610.53	43.78
C18:1_c9_(n-9c)	14795.86	561.97	16524.87	133.68	16250.94	768.60	14237.46	165.84	16100.14	275.27	19171.93	248.26	17605.61	275.82	16684.12	1318.76	15817.91	209.99	14167.25	958.10
C18:1_c10	0.00	0.00	0.00	0.00	0.00	0.00	384.81	63.59	0.00	0.00	0.00	0.00	0.00	0.00	0.00	0.00	0.00	0.00	0.00	0.00
C18:1_c11	477.15	11.75	473.27	4.88	401.39	39.18	495.06	47.62	507.73	12.48	519.25	3.20	449.77	9.94	337.68	20.76	327.90	5.24	426.79	31.73
C18:1_c12	177.35	5.34	182.60	2.37	211.32	13.22	342.45	5.78	369.80	12.10	97.56	1.47	111.86	3.91	134.89	19.96	115.79	2.46	204.09	14.19
C18:1_c13	73.59	3.25	80.45	3.15	54.56	10.18	74.93	0.70	83.21	3.39	157.78	134.19	74.79	6.58	85.47	2.80	88.00	0.22	61.89	1.54
C18:1_c14	340.99	9.26	274.26	4.22	274.07	34.48	336.19	6.75	305.03	9.81	225.55	151.03	384.68	6.29	518.23	31.88	501.21	1.55	282.50	13.54
C18:1_c15	86.63	5.11	74.67	30.81	148.11	48.37	72.64	4.27	70.33	1.43	113.12	1.73	102.42	5.68	147.82	15.28	86.52	66.39	45.27	29.59
C18:1_c16	102.91	3.86	62.27	2.01	76.37	10.08	86.78	2.13	89.68	8.42	96.51	3.58	117.76	3.35	177.58	14.49	190.36	27.61	76.02	7.56
C18:2 _t7_c9	60.12	1.53	41.64	5.75	23.61	4.59	46.72	17.92	37.58	8.79	60.03	22.88	42.52	3.24	71.44	0.75	63.73	12.27	49.20	7.80
C18:2 _c9_t11	58.68	3.91	30.22	3.22	18.69	5.00	16.23	2.53	17.68	2.68	27.94	35.66	73.96	4.92	121.97	6.56	114.26	10.28	36.74	2.74
C18:2_t10_c12	19.13	2.98	19.23	2.62	24.24	5.94	76.14	100.25	19.29	1.57	175.76	1.16	193.33	19.35	31.73	5.90	23.37	5.62	15.81	6.75
C18:2n6t (t9_t12)	183.11	6.32	181.05	24.20	232.37	9.67	187.69	6.23	210.06	4.98	0.00	0.00	226.60	22.38	489.78	32.22	483.14	5.12	180.83	9.02
C18:2_c9_t13	16.24	4.72	69.72	91.56	0.00	0.00	17.20	1.14	18.70	1.92	8.72	0.46	11.75	4.65	0.00	0.00	0.00	0.00	18.58	1.73
C18:2_t9_c13	128.06	5.83	124.32	3.98	57.86	10.68	126.48	13.44	133.56	10.16	89.04	4.37	80.70	7.08	81.95	13.15	71.05	0.49	142.59	10.44
C18:2_t8_c12	73.15	1.79	74.69	4.70	104.14	6.79	80.49	4.62	90.24	2.40	81.45	2.43	97.22	5.63	198.46	13.03	196.06	5.87	79.59	8.81
C18:2_t8_c13	90.25	4.13	74.22	3.26	108.62	37.76	81.80	6.54	89.74	3.44	82.81	5.16	99.47	8.90	145.52	12.09	146.88	0.67	80.34	4.91
C18:2_c9_t12	44.90	4.92	32.37	2.14	22.59	2.77	41.23	36.08	27.97	1.96	151.36	177.23	47.60	1.69	95.14	10.10	107.99	0.53	39.31	1.07
C18:2_t9_c12	253.80	5.61	149.41	7.21	125.86	7.76	52.75	4.60	71.97	3.60	343.96	16.53	382.03	2.67	544.49	36.14	528.24	9.46	175.16	12.43
C18:2n6c (c9_c12)	1135.67	34.37	1346.94	16.00	2290.03	130.85	1871.15	31.31	2093.00	41.72	1427.64	10.92	1387.10	32.38	1322.56	207.15	1121.22	1.09	1465.10	86.57
C18:2_t11_c15	0.00	0.00	0.00	0.00	0.00	0.00	23.78	2.63	0.00	0.00	0.00	0.00	0.00	0.00	0.00	0.00	0.00	0.00	4.19	0.62
C18:2_c9_c15	35.37	2.37	38.86	0.59	30.20	1.46	20.38	13.77	39.59	0.71	37.29	2.26	44.88	4.10	47.27	9.45	41.99	3.64	23.66	14.16
C18:3_c6_c9_c12	22.08	0.76	15.87	3.65	17.18	2.60	51.21	63.50	16.95	1.38	20.17	3.82	24.04	1.93	35.94	6.64	34.24	3.50	19.52	2.04
C20:0+C18:3_t9_t12_t15	153.60	4.73	149.01	3.48	222.74	13.38	84.49	61.51	119.92	2.42	201.47	0.94	173.73	7.48	249.39	44.76	200.92	9.52	140.06	6.66
C18:3_t9_t12_c15	0.00	0.00	0.00	0.00	0.00	0.00	24.70	2.29	0.00	0.00	0.00	0.00	0.00	0.00	24.28	6.90	0.00	0.00	0.00	0.00
C18:3_t9_c12_t15	0.00	0.00	0.00	0.00	0.00	0.00	0.00	0.00	0.00	0.00	0.00	0.00	0.00	0.00	0.00	0.00	0.00	0.00	0.00	0.00
C18:3n6 (c6_c9_c12)	24.69	1.43	24.49	3.16	25.64	2.88	25.81	2.40	33.66	2.29	22.49	2.22	28.99	2.81	25.67	4.45	17.71	2.64	27.89	2.48

**Table S1** supplementary materials. Fatty acid contend in studied cheese [mg 100g^{-1} of fat content]	S1: Camembert l'aromatique		S2: Camembert de Caractere. Roi du Chateau		S3: BucheFondante		S4: Gorgonzola Cremoso		S5: Gorgonzola Intense		S6: Cow mountain cheese non smoked		S7: Cow mountain cheese smoked		S8: Sheep mountain cheese “oscypek”non smoked		S9: Sheep mountain cheese “oscypek” smoked		S10: L'amidu Chambertin	
	x	SD	X	SD	X	SD	X	SD	X	SD	X	SD	X	SD	X	SD	X	SD	X	SD
C18:3_c9_t12_t15+c9_c12_t15	0.00	0.00	0.00	0.00	0.00	0.00	0.00	0.00	7.39	1.62	13.25	1.33	26.79	3.00	26.33	8.34	16.59	1.12	14.64	1.56
C18:3_c9_t12_c15	10.56	0.66	21.46	3.96	26.42	0.91	35.70	4.83	0.00	0.00	140.06	2.51	125.51	6.14	24.81	8.83	26.81	14.79	103.75	13.26
C18:3_t9_c12_c15	104.55	4.24	127.50	5.10	0.00	0.00	93.04	3.04	89.66	5.72	47.76	1.13	39.23	4.80	27.70	4.99	28.09	12.98	117.72	14.98
C20:1	45.43	2.87	47.68	2.81	58.81	7.56	118.76	141.86	40.53	4.07	44.90	2.12	4.06	0.96	28.00	3.52	28.15	7.54	42.13	2.94
C18:3n3 (c9_c12_c15)	437.88	14.02	404.96	1.54	540.82	31.18	310.25	12.66	344.65	5.59	923.50	9.31	1026.24	18.40	1269.90	169.45	1101.08	31.14	402.83	25.23
CLA_c9_t11+t9_c11	703.63	26.97	534.17	8.73	592.30	23.42	403.97	6.42	443.49	7.22	872.28	22.63	866.17	23.56	2204.36	228.34	2436.21	52.70	719.59	48.06
CLA_c11_t13	4.74	0.26	0.00	0.00	51.61	3.72	27.23	1.29	25.92	4.39	58.34	3.82	49.19	4.76	111.08	10.79	0.00	0.00	31.42	3.18
CLA_t10_c12	34.69	5.33	31.68	4.21	0.00	0.00	0.00	0.00	27.51	2.31	0.00	0.00	0.00	0.00	94.99	2.62	72.33	1.57	38.36	2.45
CLA_c8_c10	44.84	6.31	22.37	2.85	0.00	0.00	0.00	0.00	0.00	0.00	72.87	5.46	74.99	3.58	107.86	8.63	53.89	73.31	29.02	3.63
CLA_c9_c11	39.60	1.59	23.48	4.81	0.00	0.00	0.00	0.00	0.00	0.00	8.24	2.72	4.58	0.97	0.00	0.00	112.24	20.33	0.00	0.00
CLA_c10_c12	2.55	0.27	0.00	0.00	0.00	0.00	0.00	0.00	0.00	0.00	0.00	0.00	0.00	0.00	0.00	0.00	4.27	0.24	0.00	0.00
CLA_c11_c13	0.00	0.00	0.00	0.00	0.00	0.00	0.00	0.00	0.00	0.00	0.00	0.00	0.00	0.00	0.00	0.00	0.00	0.00	0.00	0.00
CLA_t11_t13	21.17	1.33	0.00	0.00	0.00	0.00	31.32	5.15	0.00	0.00	20.43	1.64	26.48	3.55	46.65	1.29	38.83	4.18	0.00	0.00
CLA_t12_t14	53.52	3.05	32.24	5.40	19.02	1.19	24.56	8.89	29.34	2.32	47.19	5.65	46.96	1.84	93.72	3.92	87.49	0.18	23.81	0.85
CLA_t9_t11+t10_t12	20.97	2.40	35.56	3.44	14.79	2.44	8.92	2.19	12.55	1.24	14.07	3.42	18.89	3.10	31.21	3.08	29.04	1.68	13.14	1.70
C18:3_c9_t11_t15	23.71	0.39	15.19	2.19	0.00	0.00	19.34	5.17	0.00	0.00	30.16	4.37	28.00	3.21	65.40	5.19	68.61	6.60	17.60	4.41
C20:2	30.49	5.01	26.26	5.98	23.81	6.49	16.44	9.25	28.10	2.35	25.58	3.63	23.43	0.73	30.09	2.37	25.15	0.46	31.22	4.71
CLA_t8_t10	0.00	0.00	0.00	0.00	0.00	0.00	0.00	0.00	0.00	0.00	0.00	0.00	0.00	0.00	0.00	0.00	15.39	5.10	0.00	0.00
CLA_t7_t9	0.00	0.00	0.00	0.00	0.00	0.00	20.63	1.39	0.00	0.00	0.00	0.00	0.00	0.00	0.00	0.00	0.00	0.00	0.00	0.00
C18:3_c9_t11_c15	47.42	1.09	36.02	1.87	52.58	7.86	29.23	13.82	23.88	2.66	47.86	1.14	33.84	26.57	176.73	8.77	186.66	8.20	44.48	2.66
C22:0	58.16	2.50	53.00	0.84	63.01	6.20	41.36	4.69	37.40	2.83	94.71	2.63	79.01	3.97	148.60	19.70	107.86	12.81	52.73	4.34
C20:3n6	69.18	2.38	73.73	2.91	30.56	6.04	92.66	0.76	98.85	6.75	61.38	1.81	62.44	3.89	26.35	1.80	26.41	0.32	87.85	8.84
C18:4_n3	14.23	1.17	16.39	1.74	0.00	0.00	11.56	6.47	10.98	0.85	17.03	0.78	20.34	1.27	9.66	1.24	8.41	2.46	17.23	2.30
C22:1n9	12.67	2.00	11.28	3.17	30.75	3.67	0.00	0.00	6.40	0.77	21.75	1.03	21.67	2.53	19.46	2.92	16.15	0.55	11.85	1.03
C20:3n3	16.14	1.39	13.27	2.54	16.21	1.34	125.62	13.64	0.00	0.00	4.50	0.35	0.00	0.00	9.84	3.05	22.21	4.52	12.07	0.27
C20:4n6	119.30	4.93	125.14	0.89	158.53	5.71	118.84	77.49	172.77	1.22	93.63	3.42	76.55	1.53	92.47	11.57	83.25	0.68	141.20	8.72
C23:0	19.94	20.94	30.63	18.75	25.47	5.01	11.94	6.20	16.92	0.86	12.79	2.71	32.88	35.44	97.61	14.34	45.39	51.65	40.67	2.29
C22:2	44.54	3.87	32.78	2.34	26.90	0.72	10.69	0.76	10.62	1.66	41.40	1.34	42.37	0.38	121.30	5.93	117.36	17.11	26.71	1.94
C20:5n3 EPA	50.90	1.30	44.27	0.69	160.77	9.71	27.13	2.24	33.55	10.65	85.13	3.16	131.86	66.94	92.09	13.70	80.29	3.48	37.88	1.31
C24:0	52.59	2.55	50.52	2.64	149.88	14.82	27.53	2.13	41.71	20.86	85.09	18.92	92.69	33.36	105.24	17.53	75.59	16.40	47.85	2.27
C24:1	7.69	3.93	34.38	45.02	12.82	3.75	14.45	11.48	14.16	13.65	13.07	0.86	13.87	1.98	17.80	9.09	58.77	53.32	39.91	49.57
C22:6n3 DHA	40.23	51.56	14.82	2.88	31.64	1.81	93.86	63.48	70.81	5.07	21.25	0.09	43.40	44.44	79.46	7.43	60.25	5.26	33.70	42.23

**Table S1** supplementary materials. Fatty acid contend in studied cheese [mg 100g^{-1} of fat content]	S10: L'amidu Chambertin		S11: Brie de Meaux		S12: Camembert de Normandie		S13: Epoisses de Bourgogne		S14: Petit Brillat Savarin		S15: Pon'tl'evegue		S16: Fromage de chevre au lait cru (Chevre Ronde)		S17: Crottin de Chavignol		S18: Le Fleuret		S19: Picodon Carte Noire	
	X	SD	X	SD	X	SD	X	SD	X	SD	X	SD	X	SD	X	SD	X	SD	X	SD
C4:0	3291.48	395.22	3202.59	383.28	4284.78	524.86	3571.59	947.14	3178.62	983.01	3697.20	871.93	3760.95	341.03	2779.36	751.82	2835.36	750.46	2386.53	446.61
C6:0	2111.26	127.47	2023.36	66.81	2291.93	204.85	2212.69	124.62	2044.17	43.41	2260.94	94.92	2611.90	89.73	2575.66	18.66	2744.66	46.73	2329.14	46.32
C8:0	1554.36	91.20	1544.56	57.33	1724.62	151.88	1642.81	84.49	1505.84	43.91	1686.85	63.38	3601.76	150.03	3472.73	50.26	3622.22	87.21	3098.15	42.12
C10:0	2893.13	179.53	2632.01	91.00	2905.55	213.63	2888.54	147.10	2634.73	69.07	2838.06	97.39	8759.62	382.24	8179.30	129.53	8586.33	199.24	7777.24	119.62
C12:0	3164.52	219.68	2871.45	92.78	3054.61	207.07	3107.25	169.68	2854.99	49.66	3013.99	114.48	3782.53	108.22	3466.21	51.64	3145.21	79.84	3436.25	69.99
C13:0_iso	37.18	3.74	32.07	3.22	54.12	4.25	32.72	0.49	29.94	3.90	51.96	1.18	39.76	3.09	31.67	2.95	28.14	3.28	31.78	2.44
C10:1	82.40	4.87	88.81	7.44	78.95	5.84	83.34	3.06	82.17	1.23	81.41	2.82	47.07	7.46	27.60	4.16	33.45	4.72	33.72	1.55
C13:0_ai	83.12	3.25	81.01	0.89	83.81	12.02	86.81	5.29	74.66	6.81	90.17	7.29	77.01	4.41	61.95	4.52	64.15	3.64	66.44	5.99
C14:0_iso	112.86	8.02	76.88	1.27	133.28	9.53	92.96	6.52	82.66	4.27	142.65	4.57	101.16	2.20	97.77	1.72	73.72	3.04	118.32	1.64
C14:0	10133.44	708.78	9312.18	284.16	9700.02	604.83	10003.52	555.02	9218.63	141.58	9981.56	389.91	8621.97	173.92	8177.57	64.50	7737.04	199.12	8835.54	211.47
C15:O_iso	235.18	15.38	203.56	8.26	303.61	18.80	214.40	14.19	192.25	4.26	303.21	5.25	214.76	5.80	192.68	7.59	184.24	4.89	233.54	11.26
C15:O_ai	518.29	34.46	410.24	13.42	573.09	37.32	449.37	31.74	470.11	6.22	541.55	22.64	349.12	5.27	331.34	5.91	287.15	10.08	388.08	12.68
C14:1_c9_(n-5)	692.66	50.79	849.30	25.70	719.84	48.91	781.64	42.48	773.93	4.06	803.43	35.74	151.77	9.60	105.06	8.09	111.39	4.27	117.60	3.05
C15:0	1072.58	83.80	970.00	37.31	1095.81	66.81	1002.18	55.28	1014.94	20.15	1069.57	40.23	839.68	16.12	853.86	17.97	735.62	17.58	1001.53	35.69
C16:0_iso	41.58	2.88	30.09	2.11	41.95	5.38	34.44	2.14	45.23	0.91	37.60	2.38	47.57	1.95	56.78	4.24	59.08	2.84	75.24	0.10
C16:0_ai	275.71	10.96	220.80	9.15	300.40	32.74	243.62	21.74	233.16	12.69	351.15	18.58	251.34	14.61	250.70	10.32	208.82	5.09	260.25	24.99
C15:1_n10_(n-5)	15.57	2.28	20.05	0.36	16.55	1.70	18.61	3.27	11.29	8.89	17.79	0.31	12.80	2.16	13.30	1.30	9.97	1.53	18.09	0.42
C16:0	24465.18	1683.70	24218.62	674.67	21784.17	1289.31	25063.58	1431.66	24415.17	403.39	23764.53	920.84	19651.38	304.44	17371.97	75.28	22720.02	663.57	21453.98	489.49
C17:O_iso	385.50	25.94	365.10	9.12	515.78	33.18	375.56	21.92	361.69	4.36	454.91	21.41	386.96	10.50	430.09	6.07	401.27	10.82	419.20	9.61
C17:O_ai	157.48	10.91	148.27	5.75	193.33	12.59	163.45	8.23	146.52	16.42	206.28	21.12	242.11	7.20	240.23	3.05	266.64	6.02	257.63	5.98
C16:1_t9	438.33	35.21	386.55	11.14	407.36	23.32	383.70	23.42	472.35	9.72	428.78	20.76	365.81	9.90	381.44	2.57	329.60	3.65	420.43	10.11
C16:1_c9_(n-7)	1045.91	72.54	1204.97	36.83	866.05	57.72	1144.45	67.12	1089.99	9.21	1081.12	51.60	457.40	15.29	316.89	7.02	491.89	19.74	406.72	11.76
C16:1_t10	39.20	3.35	32.45	0.91	130.08	6.59	36.72	1.93	33.92	1.93	72.63	1.37	23.08	3.61	28.29	7.18	26.22	2.69	28.46	1.46
C16:1_t11_+_t12	30.94	7.40	35.94	6.15	27.45	2.73	34.89	0.60	34.67	3.21	39.11	2.49	13.46	3.90	13.53	10.46	15.65	2.86	14.22	10.45
C17:0	617.91	42.08	599.31	16.73	638.39	38.08	606.83	33.46	637.27	9.19	647.47	28.03	635.56	18.97	658.26	7.07	644.90	16.91	788.52	18.02
C18:0_iso	53.77	3.82	47.26	3.38	55.83	4.02	48.24	1.11	54.52	3.93	64.09	1.86	52.62	1.34	58.85	5.33	46.15	3.91	61.60	4.50
C18_0_ai	31.90	5.56	34.01	1.78	30.38	3.45	30.89	1.78	33.61	1.96	34.71	1.12	37.46	1.24	42.97	5.56	40.31	1.33	40.83	0.86
C17:1_c9	27.49	2.57	30.64	1.74	66.07	4.05	28.13	3.09	30.39	4.67	50.85	2.71	31.41	0.65	29.10	0.33	13.34	0.68	34.06	1.20
C17:1_c10_(n-7)	165.70	10.17	187.73	3.62	162.04	11.67	182.88	13.20	177.18	1.86	188.44	11.42	189.97	3.52	157.90	2.60	181.70	6.13	214.74	4.25
C18:0	8046.00	553.62	8075.75	214.52	9972.91	564.57	7745.66	444.37	7488.86	133.48	9755.31	360.30	8642.88	242.00	11787.32	83.75	10864.09	325.33	9000.09	223.11
C18:1_t6	19.93	1.46	21.89	1.33	20.28	2.36	20.03	3.13	18.35	4.19	17.86	1.74	12.63	7.70	23.47	7.22	14.49	8.01	11.46	7.48
C18:1_t7	22.10	2.72	23.65	1.02	19.78	2.17	25.38	4.47	29.25	4.19	18.24	4.11	20.13	4.17	25.09	4.55	27.64	6.67	19.35	2.06

**Table S1** supplementary materials. Fatty acid contend in studied cheese [mg 100g^{-1} of fat content]	S10: L'amidu Chambertin		S11: Brie de Meaux		S12: Camembert de Normandie		S13: Epoisses de Bourgogne		S14: Petit Brillat Savarin		S15: Pon'tl'evegue		S16: Fromage de chevre au lait cru (Chevre Ronde)		S17: Crottin de Chavignol		S18: Le Fleuret		S19: Picodon Carte Noire	
	X	SD	X	SD	X	SD	X	SD	X	SD	X	SD	X	SD	X	SD	X	SD	X	SD
C18:1_t8	213.57	14.41	236.03	9.27	185.12	8.90	211.41	14.31	186.96	10.77	180.00	9.50	166.74	8.51	184.70	2.18	213.56	11.35	139.39	2.33
C18:1_t9	224.56	19.81	240.44	3.58	196.56	12.13	233.16	15.58	199.71	6.36	216.01	2.76	199.41	12.21	228.56	4.37	255.07	6.03	190.11	11.62
C18:1_t10	0.00	0.00	452.57	19.72	0.00	0.00	315.21	23.41	328.93	9.26	2145.85	177.42	289.91	3.90	0.00	0.00	348.78	15.12	181.82	8.02
C18:1_t11	1767.03	119.16	1116.28	25.77	3193.70	184.43	1575.98	127.16	911.05	4.59	2034.77	29.00	937.58	35.57	1362.83	11.16	988.99	28.63	673.91	17.48
C18:1_t12	253.98	20.65	342.69	10.28	209.61	11.07	267.93	16.12	262.07	2.70	214.74	9.67	184.70	7.14	300.82	5.20	294.65	6.16	167.82	3.33
C18:1_t13+t14	610.53	43.78	796.46	28.74	637.15	38.85	659.37	41.72	663.90	8.32	585.26	18.44	401.05	9.89	730.80	4.28	606.62	20.71	359.88	11.82
C18:1_c9_(n-9c)	14167.25	958.10	14400.58	383.24	13902.08	844.02	15319.55	1011.50	12552.41	103.29	15266.03	749.86	14692.27	384.93	16695.09	113.75	17027.75	537.83	14047.47	345.16
C18:1_c10	0.00	0.00	0.00	0.00	0.00	0.00	0.00	0.00	0.00	0.00	0.00	0.00	14.58	1.03	0.00	0.00	0.00	0.00	0.00	0.00
C18:1_c11	426.79	31.73	451.60	14.66	367.36	21.43	515.04	30.82	416.46	0.73	440.85	19.88	352.55	8.89	356.55	8.47	301.45	13.19	366.15	18.84
C18:1_c12	204.09	14.19	299.04	9.62	105.60	7.12	215.96	10.98	231.71	2.23	152.27	6.12	144.35	1.35	207.30	3.35	220.87	7.94	137.76	4.61
C18:1_c13	61.89	1.54	80.61	3.92	62.12	2.81	76.91	5.87	68.77	1.87	69.79	4.12	43.67	1.28	54.43	2.28	53.37	1.98	42.92	0.93
C18:1_c14	282.50	13.54	369.40	21.12	329.57	17.65	295.67	17.39	299.27	8.83	284.21	12.78	218.04	2.85	392.56	2.29	319.64	11.21	207.45	5.76
C18:1_c15	45.27	29.59	91.69	6.93	80.75	3.11	79.98	8.10	80.57	7.36	91.24	6.84	145.97	3.75	76.32	39.07	178.17	6.77	147.29	32.13
C18:1_c16	76.02	7.56	88.35	7.93	111.34	3.78	91.28	4.38	75.08	2.80	79.89	4.54	0.00	0.00	125.65	0.20	0.00	0.00	49.28	2.72
C18:2 _t7_c9	49.20	7.80	56.38	3.58	90.14	3.28	57.05	2.62	44.74	4.19	66.04	1.87	20.23	11.78	29.99	2.54	30.01	1.99	17.51	7.32
C18:2 _c9_t11	36.74	2.74	32.33	6.32	97.53	2.42	46.31	1.48	23.01	4.37	51.32	4.81	20.29	2.40	21.58	16.11	0.00	0.00	16.27	1.86
C18:2_t10_c12	15.81	6.75	21.78	2.49	17.80	2.13	25.84	1.31	15.48	0.63	0.00	0.00	15.28	2.03	17.78	3.41	15.00	1.24	0.00	0.00
C18:2n6t (t9_t12)	180.83	9.02	233.74	10.53	189.74	8.81	214.74	11.24	168.25	5.74	162.07	6.43	159.00	3.83	275.66	2.14	227.05	5.86	132.83	2.36
C18:2_c9_t13	18.58	1.73	18.10	5.15	13.47	1.17	16.35	1.83	18.35	4.11	19.43	4.64	5.19	0.40	4.84	1.64	0.00	0.00	135.33	0.47
C18:2_t9_c13	142.59	10.44	149.80	4.55	90.06	15.80	142.13	0.95	115.89	3.16	138.62	6.49	69.03	6.21	60.79	6.14	49.24	0.91	54.27	1.07
C18:2_t8_c12	79.59	8.81	102.90	9.70	76.26	7.03	89.15	2.86	75.85	2.92	65.69	3.65	69.86	2.22	127.87	5.92	101.17	3.71	51.49	5.17
C18:2_t8_c13	80.34	4.91	107.71	21.95	85.05	5.93	85.34	4.31	76.91	1.14	77.53	6.26	67.13	5.22	111.23	5.15	91.30	9.18	63.84	3.79
C18:2_c9_t12	39.31	1.07	35.04	2.29	65.94	3.73	38.67	2.85	25.60	1.48	45.10	3.20	21.02	4.18	25.02	1.67	15.45	1.90	17.64	2.12
C18:2_t9_c12	175.16	12.43	137.60	2.40	399.35	22.64	204.52	5.09	127.76	4.26	249.31	8.90	99.12	13.14	253.94	12.39	42.51	31.20	74.92	5.54
C18:2n6c (c9_c12)	1465.10	86.57	1546.48	36.15	939.18	71.83	1630.54	156.50	1497.15	3.08	1068.87	47.19	1915.13	49.72	2320.75	11.20	1971.04	73.20	1768.43	47.02
C18:2_t11_c15	4.19	0.62	0.00	0.00	0.00	0.00	0.00	0.00	0.00	0.00	0.00	0.00	0.00	0.00	0.00	0.00	0.00	0.00	0.00	0.00
C18:2_c9_c15	23.66	14.16	39.93	6.79	32.46	4.91	40.37	3.70	34.79	5.09	26.83	15.94	15.88	11.08	45.28	5.42	0.00	0.00	17.65	2.70
C18:3_c6_c9_c12	19.52	2.04	17.55	0.12	26.04	1.95	15.78	5.44	19.80	3.98	16.16	0.87	0.00	0.00	48.05	3.47	0.00	0.00	0.00	0.00
C20:0+C18:3_t9_t12_t15	140.06	6.66	127.60	5.41	144.76	14.07	122.16	7.46	117.85	2.65	151.49	7.89	200.50	6.14	216.34	1.96	317.17	11.71	273.97	8.92
C18:3_t9_t12_c15	0.00	0.00	0.00	0.00	0.00	0.00	13.18	2.77	0.00	0.00	0.00	0.00	0.00	0.00	0.00	0.00	0.00	0.00	0.00	0.00
C18:3_t9_c12_t15	0.00	0.00	0.00	0.00	0.00	0.00	0.00	0.00	0.00	0.00	0.00	0.00	0.00	0.00	0.00	0.00	0.00	0.00	0.00	0.00
C18:3n6 (c6_c9_c12)	27.89	2.48	22.82	4.58	16.25	0.69	26.01	3.46	32.59	6.21	19.80	1.70	28.83	3.75	20.35	1.80	24.58	0.22	19.96	3.04

**Table S1** supplementary materials. Fatty acid contend in studied cheese [mg 100g^{-1} of fat content]	S10: L'amidu Chambertin		S11: Brie de Meaux		S12: Camembert de Normandie		S13: Epoisses de Bourgogne		S14: Petit Brillat Savarin		S15: Pon'tl'evegue		S16: Fromage de chevre au lait cru (Chevre Ronde)		S17: Crottin de Chavignol		S18: Le Fleuret		S19: Picodon Carte Noire	
	X	SD	X	SD	X	SD	X	SD	X	SD	X	SD	X	SD	X	SD	X	SD	X	SD
C18:3_c9_t12_t15+c9_c12_t15	14.64	1.56	0.00	0.00	0.00	0.00	5.33	1.03	0.00	0.00	15.84	2.88	15.00	1.30	16.45	2.30	4.89	1.01	0.00	0.00
C18:3_c9_t12_c15	103.75	13.26	109.16	2.73	96.73	6.06	17.93	3.03	0.00	0.00	117.04	14.74	15.09	2.30	12.80	2.57	12.40	5.93	16.10	3.05
C18:3_t9_c12_c15	117.72	14.98	73.92	50.37	95.83	9.60	107.85	15.05	95.51	3.18	105.10	2.46	15.74	2.88	11.58	0.44	20.99	2.04	17.89	7.38
C20:1	42.13	2.94	43.76	2.42	32.17	3.14	42.91	6.15	49.63	11.70	39.46	4.50	46.48	8.54	36.16	1.71	50.78	9.36	45.33	4.71
C18:3n3 (c9_c12_c15)	402.83	25.23	398.49	12.90	617.46	57.29	451.55	41.63	372.27	16.03	419.91	15.31	471.38	16.07	1500.03	22.98	271.47	16.02	570.82	16.91
CLA_c9_t11+t9_c11	719.59	48.06	549.82	19.04	1169.81	73.06	702.42	44.27	430.48	9.26	772.65	30.78	498.63	14.30	569.20	23.85	483.96	10.16	325.19	11.26
CLA_c11_t13	31.42	3.18	30.77	1.62	42.74	7.10	29.89	2.44	33.15	4.92	28.78	15.11	50.93	6.77	59.45	3.13	60.94	2.89	70.05	1.39
CLA_t10_c12	38.36	2.45	0.00	0.00	10.60	0.59	32.67	4.66	36.84	3.02	39.39	4.44	47.82	4.08	5.91	0.29	0.00	0.00	73.57	2.43
CLA_c8_c10	29.02	3.63	19.87	3.41	70.64	8.96	35.46	5.29	19.85	1.51	43.75	2.86	15.60	1.48	51.86	1.49	0.00	0.00	3.13	0.26
CLA_c9_c11	0.00	0.00	0.00	0.00	0.00	0.00	28.71	3.31	5.00	0.51	0.00	0.00	0.00	0.00	5.35	1.25	0.00	0.00	0.00	0.00
CLA_c10_c12	0.00	0.00	0.00	0.00	0.00	0.00	0.00	0.00	2.64	0.52	0.00	0.00	0.00	0.00	0.00	0.00	0.00	0.00	0.00	0.00
CLA_c11_c13	0.00	0.00	22.34	5.29	0.00	0.00	0.00	0.00	0.00	0.00	0.00	0.00	0.00	0.00	0.00	0.00	10.31	0.52	0.00	0.00
CLA_t11_t13	0.00	0.00	17.41	0.36	22.12	4.61	0.00	0.00	0.00	0.00	0.00	0.00	0.00	0.00	28.64	7.59	0.00	0.00	0.00	0.00
CLA_t12_t14	23.81	0.85	39.02	3.64	57.78	4.53	39.42	0.41	32.18	2.05	31.82	2.46	18.82	0.31	35.76	2.14	0.00	0.00	11.97	0.54
CLA_t9_t11+t10_t12	13.14	1.70	19.87	4.41	21.98	3.40	15.58	2.32	16.61	5.19	0.00	0.00	14.48	3.41	11.01	1.31	0.00	0.00	8.64	0.53
C18:3_c9_t11_t15	17.60	4.41	16.63	3.89	39.03	2.27	19.24	1.90	15.02	2.00	28.04	6.85	14.49	3.64	21.20	1.24	0.00	0.00	0.00	0.00
C20:2	31.22	4.71	25.76	2.68	17.80	1.81	26.85	1.13	25.79	4.77	24.67	4.32	17.16	1.24	23.94	3.65	20.14	3.84	24.59	1.40
CLA_t8_t10	0.00	0.00	0.00	0.00	0.00	0.00	0.00	0.00	0.00	0.00	0.00	0.00	0.00	0.00	0.00	0.00	0.00	0.00	0.00	0.00
CLA_t7_t9	0.00	0.00	0.00	0.00	0.00	0.00	0.00	0.00	0.00	0.00	0.00	0.00	0.00	0.00	0.00	0.00	0.00	0.00	8.43	0.61
C18:3_c9_t11_c15	44.48	2.66	37.69	2.05	72.26	1.62	42.89	2.06	39.28	2.82	52.93	1.03	48.80	2.87	137.92	3.72	42.03	4.61	44.55	3.25
C22:0	52.73	4.34	47.14	0.66	63.30	4.98	45.77	5.88	46.93	4.35	65.85	2.18	59.58	8.13	67.17	4.66	104.30	4.01	105.08	7.56
C20:3n6	87.85	8.84	77.00	3.44	55.50	3.16	74.10	5.13	87.95	0.16	72.29	4.94	23.13	1.34	20.75	2.43	31.58	0.70	24.39	3.25
C18:4_n3	17.23	2.30	13.37	0.41	15.16	2.43	12.02	0.41	14.46	1.59	16.80	1.37	0.00	0.00	0.00	0.00	8.93	2.04	7.86	1.20
C22:1n9	11.85	1.03	10.81	2.17	17.14	1.94	10.73	3.90	10.60	2.20	12.45	1.89	11.32	5.13	22.87	1.11	22.91	11.78	14.48	2.49
C20:3n3	12.07	0.27	5.21	1.19	5.01	1.64	16.21	3.01	14.89	0.68	19.73	1.44	10.38	2.99	11.85	0.57	0.00	0.00	13.11	9.28
C20:4n6	141.20	8.72	121.38	5.09	88.57	4.80	130.68	5.91	145.06	1.00	122.97	8.06	156.72	2.41	143.42	8.09	184.98	7.46	147.23	0.54
C23:0	40.67	2.29	23.79	11.41	35.24	26.74	31.38	8.72	32.34	10.87	20.36	21.14	14.17	11.49	18.90	19.24	27.77	13.06	8.43	4.98
C22:2	26.71	1.94	25.44	2.63	66.84	3.83	26.21	2.93	25.08	1.33	43.43	2.38	33.26	2.38	27.39	2.65	10.26	0.52	29.39	2.24
C20:5n3 EPA	37.88	1.31	40.02	2.05	73.73	5.87	39.40	1.41	38.21	3.34	54.05	5.43	44.86	2.06	69.29	2.79	36.85	2.43	52.68	1.86
C24:0	47.85	2.27	42.54	2.77	58.07	4.61	42.71	3.55	42.58	3.58	60.35	5.78	37.06	2.83	34.58	3.24	42.65	3.92	63.04	2.96
C24:1	39.91	49.57	38.45	49.72	32.24	34.79	6.71	5.60	11.99	1.33	13.50	4.32	13.45	1.66	12.19	1.30	10.81	2.08	19.45	2.20
C22:6n3 DHA	33.70	42.23	65.12	47.85	20.45	1.05	7.34	3.18	34.09	49.93	10.79	4.58	33.31	1.88	63.35	4.37	23.59	1.72	44.91	6.25

**Table S1** supplementary materials. Fatty acid contend in studied cheese [mg $100g^{-1}$ of fat content]	S20: Sainte Maure de Touraine. Hardy Affineur		S21: Selles sur Cher. Appellation d'origine protégée		S22: English Cheddar Farmhouse		S23: EmmentalerFrancais		S24: RacletteBadozPrest		S25: Tette de Moine		S26: Tomme de SavoieYenn		S27: TommeChevre		S28: Appenzeller extra noir		S29: Beaufort	
	X	SD	X	SD	X	SD	X	SD	X	SD	X	SD	X	SD	X	SD	X	SD	X	SD
C4:0	2452.16	438.53	2681.09	1027.99	3347.64	354.36	3384.39	384.49	3376.53	444.29	3309.26	966.51	3297.88	421.75	3348.86	420.50	3514.22	444.66	3790.18	522.76
C6:0	2131.96	54.75	2520.90	57.96	2193.57	80.51	2267.40	7.64	2388.41	42.66	2137.54	76.02	2138.41	69.25	2570.46	138.93	2305.45	35.99	2183.67	4.48
C8:0	2804.25	67.89	3573.38	86.09	1622.85	47.57	1707.13	16.57	1851.88	35.75	1618.84	123.76	1612.18	54.72	3582.94	166.74	1728.32	23.59	1569.13	9.49
C10:0	6940.99	111.66	8807.14	236.43	2706.71	60.11	2860.03	11.08	3241.34	58.23	2786.43	393.99	2750.30	85.19	8411.29	364.91	2794.21	36.95	2429.36	21.22
C12:0	3063.01	26.73	3991.89	97.75	3150.96	49.34	3040.64	31.63	3495.51	59.87	2766.90	48.83	2944.17	84.43	3531.88	167.31	2921.40	59.85	2559.63	56.58
C13:0_iso	26.16	2.08	20.57	8.97	33.14	4.45	31.51	4.99	34.55	1.67	36.75	2.73	48.23	2.61	26.09	4.89	39.34	1.12	51.67	2.26
C10:1	43.45	2.28	53.66	6.47	93.41	2.49	85.68	0.46	95.04	1.42	83.95	4.03	77.48	0.80	32.99	3.25	93.84	4.08	65.54	3.12
C13:0_ai	74.23	4.28	95.30	2.57	88.35	4.97	91.15	8.21	94.06	5.06	77.54	7.20	83.48	6.51	62.24	6.07	97.00	13.12	61.94	1.45
C14:0_iso	62.62	1.63	87.70	2.53	87.22	1.74	107.37	1.90	112.99	1.76	133.24	3.10	130.00	1.78	90.31	2.77	100.39	3.73	128.87	2.50
C14:0	7620.55	49.34	8043.42	182.69	9416.26	148.21	9796.49	149.46	10758.55	179.45	9636.61	144.31	9898.91	291.88	8056.05	378.47	9715.77	211.06	8854.98	237.34
C15:O_iso	178.82	3.49	169.40	5.60	226.71	2.64	218.98	4.24	217.72	5.23	276.81	3.79	284.90	10.57	195.00	9.28	225.50	5.27	268.47	4.63
C15:O_ai	282.37	18.89	288.04	10.02	413.56	3.61	395.26	11.96	425.64	5.35	493.35	17.95	537.47	16.57	310.02	16.93	439.25	13.33	482.64	9.65
C14:1_c9_(n-5)	151.60	13.19	145.60	5.94	855.61	14.97	840.80	10.91	867.28	16.18	858.23	47.40	778.06	21.19	109.13	3.08	931.87	21.59	645.68	24.89
C15:0	770.02	12.14	803.95	22.90	976.25	24.93	1031.50	28.79	1066.38	21.24	1059.56	23.81	1055.41	32.21	757.84	32.99	1003.81	30.73	892.55	39.37
C16:0_iso	43.68	2.75	69.61	0.38	30.16	0.76	34.56	2.34	29.35	1.92	44.80	1.48	50.60	1.33	46.43	3.91	46.50	1.66	83.38	1.36
C16:0_ai	211.12	1.53	245.53	2.32	202.03	26.87	250.17	6.83	292.53	5.65	270.22	15.85	297.15	25.91	229.45	27.40	211.31	39.17	254.56	7.13
C15:1_n10_(n-5)	6.93	7.32	8.68	3.01	25.25	4.04	20.27	3.94	13.38	7.81	15.10	6.66	13.67	7.41	9.16	3.44	21.02	1.61	13.60	1.87
C16:0	23408.49	130.15	18284.26	401.93	25983.32	373.78	26082.07	405.46	27344.39	479.53	27364.44	355.16	22644.03	663.69	18855.93	893.08	23612.10	486.91	21815.89	597.36
C17:O_iso	429.06	3.74	527.45	16.23	360.59	10.30	332.40	4.80	320.06	7.54	413.32	9.69	511.64	13.93	424.38	20.84	472.88	14.44	537.78	18.02
C17:O_ai	242.11	3.18	200.09	5.72	161.85	24.10	148.79	14.75	163.33	16.53	157.86	12.40	201.43	17.84	248.29	11.14	188.28	4.82	233.88	4.39
C16:1_t9	339.94	1.81	394.59	14.64	356.54	16.26	326.00	6.87	372.75	4.10	398.23	7.72	429.43	14.78	362.05	17.22	341.35	7.37	363.27	10.01
C16:1_c9_(n-7)	594.84	9.94	456.40	9.37	1391.55	11.55	1397.58	22.32	1318.50	25.95	1296.97	50.81	1104.35	28.68	439.59	21.90	1207.94	30.85	947.90	28.18
C16:1_t10	20.21	1.47	45.26	0.41	55.20	3.53	62.15	4.02	35.87	2.17	64.19	2.14	65.59	2.04	26.39	0.95	130.58	5.57	105.13	4.54
C16:1_t11_+_t12	18.01	3.49	22.92	21.78	37.80	2.22	37.68	1.82	36.08	3.94	33.95	1.29	32.26	4.10	18.87	6.47	34.95	1.01	29.17	2.75
C17:0	542.51	4.89	647.62	16.54	557.05	11.66	605.25	11.00	632.13	13.56	665.20	10.33	645.08	14.63	639.95	30.80	600.46	14.24	621.27	14.34
C18:0_iso	38.60	1.94	61.12	2.84	49.17	2.65	57.90	4.10	52.19	1.36	68.62	3.20	65.22	2.34	54.19	2.03	63.97	18.37	70.17	4.75
C18_0_ai	44.22	3.14	43.48	5.40	34.37	2.17	32.21	0.35	36.50	2.29	33.08	1.05	30.00	3.70	37.27	0.85	27.25	1.14	34.43	1.72
C17:1_c9	26.25	1.34	20.40	3.21	59.62	1.49	52.13	2.01	30.17	1.36	60.31	2.27	49.86	2.18	33.57	1.56	55.83	3.12	53.24	2.03
C17:1_c10_(n-7)	181.57	3.45	175.84	3.40	196.42	5.22	207.47	2.24	197.48	6.60	219.18	0.70	199.28	5.32	209.12	10.27	199.55	6.11	196.17	6.45
C18:0	8202.41	58.71	7291.86	180.32	7966.78	127.13	7686.42	128.04	7590.88	133.47	7856.46	170.04	9111.80	272.25	9928.57	488.90	8210.40	185.87	10569.10	270.91
C18:1_t6	16.73	10.69	16.34	10.48	19.82	3.56	12.52	9.51	18.34	1.84	13.07	6.77	21.65	2.53	15.93	0.76	13.85	10.83	19.25	1.17
C18:1_t7	25.98	3.14	27.02	1.74	20.71	4.73	14.23	8.99	16.55	2.84	17.18	2.08	25.00	4.85	19.44	1.81	22.55	1.01	20.44	1.07

**Table S1** supplementary materials. Fatty acid contend in studied cheese [mg 100g^{-1} of fat content]	S20: Sainte Maure de Touraine. Hardy Affineur		S21: Selles sur Cher. Appellation d'origine protégée		S22: English Cheddar Farmhouse		S23: EmmentalerFrancais		S24: RacletteBadozPrest		S25: Tette de Moine		S26: Tomme de SavoieYenn		S27: TommeChevre		S28: Appenzeller extra noir		S29: Beaufort	
	X	SD	X	SD	X	SD	X	SD	X	SD	X	SD	X	SD	X	SD	X	SD	X	SD
C18:1_t8	267.61	4.12	276.05	4.67	179.79	7.50	153.87	3.73	175.25	10.32	181.62	12.12	235.28	5.58	195.06	9.61	215.08	5.44	215.00	8.32
C18:1_t9	304.70	0.27	307.85	9.07	203.04	7.93	185.04	9.32	222.22	3.84	211.37	10.21	249.38	17.54	235.18	10.09	225.22	5.38	230.36	2.20
C18:1_t10	401.61	8.23	617.20	25.77	0.00	0.00	1436.88	75.39	257.53	11.93	0.00	0.00	0.00	0.00	305.20	17.50	0.00	0.00	0.00	0.00
C18:1_t11	961.44	6.34	2004.12	56.06	1424.75	29.72	1405.66	29.37	794.03	19.48	1572.93	23.79	2427.05	72.60	1212.36	60.34	3105.80	77.22	3409.43	82.98
C18:1_t12	290.01	1.96	354.46	11.65	233.10	0.79	201.46	4.35	241.76	8.10	176.14	12.68	262.65	7.11	246.36	12.84	236.92	8.29	251.13	3.58
C18:1_t13+t14	518.16	9.55	992.19	23.91	660.77	12.53	518.77	9.45	566.33	8.34	437.10	9.74	642.27	20.45	550.46	26.21	621.89	12.88	715.05	17.89
C18:1_c9_(n-9c)	17706.02	139.75	12278.14	351.20	14972.26	229.31	15109.55	236.77	15363.01	304.86	15130.69	467.11	17901.57	609.03	16388.80	830.11	16055.86	380.50	16927.77	434.03
C18:1_c10	0.00	0.00	496.91	49.06	0.00	0.00	0.00	0.00	0.00	0.00	16.29	0.73	0.00	0.00	0.00	0.00	0.00	0.00	0.00	0.00
C18:1_c11	464.16	8.73	433.72	10.27	396.59	5.00	428.84	10.91	483.12	12.20	376.00	11.72	518.48	16.94	378.23	18.12	386.72	6.77	403.64	10.17
C18:1_c12	190.36	1.64	613.43	14.49	156.66	5.11	160.18	2.32	216.43	4.24	102.68	10.59	177.59	7.58	192.18	6.25	120.51	3.37	135.11	3.21
C18:1_c13	58.33	0.43	77.56	2.90	68.58	1.89	70.41	2.82	60.99	5.31	57.90	2.78	74.15	3.36	49.64	4.15	74.53	2.61	72.58	2.29
C18:1_c14	247.24	3.58	362.59	10.81	298.25	6.53	264.33	8.65	266.13	0.56	209.45	9.43	332.14	10.71	267.17	14.89	314.46	4.04	405.61	5.71
C18:1_c15	144.26	1.63	107.42	6.36	72.10	5.73	69.00	3.67	59.24	26.65	94.80	57.06	82.11	2.12	62.55	38.29	70.92	2.82	94.63	4.21
C18:1_c16	0.00	0.00	256.52	67.68	99.05	3.79	91.49	10.81	4.84	0.39	54.00	1.47	102.20	4.95	82.21	6.51	144.02	40.36	130.91	2.17
C18:2 _t7_c9	35.83	15.66	31.80	5.03	52.36	2.94	49.28	0.77	34.76	0.88	54.89	5.69	65.46	4.88	20.66	6.17	67.08	1.63	44.99	6.97
C18:2 _c9_t11	0.00	0.00	56.62	4.89	40.80	2.14	47.82	2.79	19.02	0.99	35.49	4.59	62.94	8.82	20.25	13.96	90.85	3.64	110.70	7.58
C18:2_t10_c12	20.63	2.44	36.39	3.60	24.56	3.19	22.05	4.67	14.94	3.17	16.26	0.73	22.39	1.93	14.93	3.00	17.40	2.74	16.44	0.74
C18:2n6t (t9_t12)	252.10	1.92	400.58	16.06	199.28	6.11	172.77	4.16	182.21	7.29	137.24	3.39	232.64	9.46	216.08	12.18	229.13	4.94	243.67	0.69
C18:2_c9_t13	2.56	0.38	11.86	0.82	18.38	4.68	16.13	3.34	15.75	1.44	15.13	3.99	11.53	0.95	5.37	0.42	5.13	0.85	7.37	1.69
C18:2_t9_c13	57.81	1.87	38.70	7.50	84.24	3.49	106.95	2.98	161.25	2.93	86.85	8.82	143.19	5.95	57.30	4.58	65.18	6.67	125.01	4.91
C18:2_t8_c12	113.05	1.41	157.05	8.58	88.70	8.07	80.27	2.14	82.23	7.35	60.23	2.56	99.29	2.38	95.80	8.86	90.19	4.46	103.74	0.71
C18:2_t8_c13	105.22	8.13	124.67	16.60	79.47	6.58	68.10	4.29	81.56	3.27	67.24	2.16	98.25	5.19	80.44	4.15	79.71	2.75	101.04	5.50
C18:2_c9_t12	29.64	1.78	49.49	3.42	29.82	8.73	29.95	2.62	55.76	49.52	30.30	1.92	52.62	6.26	27.53	5.02	61.86	3.48	65.38	1.65
C18:2_t9_c12	42.89	3.44	379.44	22.49	188.60	6.23	223.39	2.50	117.73	3.76	174.75	7.72	278.09	11.05	178.43	16.10	408.27	13.61	493.41	11.75
C18:2n6c (c9_c12)	2327.41	17.16	2477.90	75.49	1219.82	7.08	1221.61	25.02	1359.25	22.72	1375.17	109.29	1737.52	74.13	2120.68	103.80	1228.55	22.32	1779.87	38.13
C18:2_t11_c15	0.00	0.00	56.89	6.64	0.00	0.00	0.00	0.00	2.72	0.15	0.00	0.00	0.00	0.00	0.00	0.00	0.00	0.00	18.08	31.32
C18:2_c9_c15	30.36	4.84	47.20	2.92	40.45	8.04	40.32	4.43	39.35	4.61	33.64	4.82	28.37	11.72	27.78	22.02	40.91	1.98	32.39	5.45
C18:3_c6_c9_c12	9.88	2.08	93.23	6.97	20.04	2.74	19.92	3.14	16.25	1.03	0.00	0.00	20.91	1.13	25.57	5.96	20.82	2.96	24.31	1.59
C20:0+C18:3_t9_t12_t15	208.70	3.93	145.70	15.23	126.79	1.31	131.23	2.07	125.54	1.04	169.87	9.25	145.79	5.85	208.23	13.85	120.22	2.06	181.05	9.94
C18:3_t9_t12_c15	7.28	1.69	29.16	2.86	0.00	0.00	0.00	0.00	0.00	0.00	0.00	0.00	0.00	0.00	0.00	0.00	0.00	0.00	0.00	0.00
C18:3_t9_c12_t15	0.00	0.00	0.00	0.00	0.00	0.00	0.00	0.00	0.00	0.00	0.00	0.00	0.00	0.00	0.00	0.00	0.00	0.00	0.00	0.00
C18:3n6 (c6_c9_c12)	23.01	3.53	22.87	0.82	21.28	2.79	14.55	5.88	22.71	8.00	23.50	1.14	35.15	0.91	21.60	4.57	18.54	2.44	25.05	2.34

**Table S1** supplementary materials. Fatty acid contend in studied cheese [mg $100g^{-1}$ of fat content]	S20: Sainte Maure de Touraine. Hardy Affineur		S21: Selles sur Cher. Appellation d'origine protégée		S22: English Cheddar Farmhouse		S23: EmmentalerFrancais		S24: RacletteBadozPrest		S25: Tette de Moine		S26: Tomme de SavoieYenn		S27: TommeChevre		S28: Appenzeller extra noir		S29: Beaufort	
	X	SD	X	SD	X	SD	X	SD	X	SD	X	SD	X	SD	X	SD	X	SD	X	SD
C18:3_c9_t12_t15+c9_c12_t15	0.00	0.00	16.92	2.32	0.00	0.00	0.00	0.00	0.00	0.00	7.83	0.98	0.00	0.00	0.00	0.00	15.71	2.35	16.69	5.68
C18:3_c9_t12_c15	21.05	5.00	0.00	0.00	113.74	14.47	16.09	3.50	103.45	7.16	17.16	0.62	15.50	1.46	10.85	0.84	16.80	1.98	16.68	1.87
C18:3_t9_c12_c15	25.80	3.88	59.29	4.51	107.21	1.66	116.90	10.39	90.10	39.86	150.44	13.56	123.59	11.08	0.00	0.00	112.59	7.36	134.61	6.32
C20:1	70.52	5.63	323.37	474.99	40.32	1.90	48.42	4.24	40.48	1.57	52.88	7.03	45.39	1.36	47.19	6.73	36.37	5.28	40.29	3.85
C18:3n3 (c9_c12_c15)	291.78	5.31	859.10	68.87	423.72	15.48	500.93	16.38	344.52	4.07	613.86	25.28	705.68	18.72	638.93	26.12	779.35	25.66	1472.69	38.90
CLA_c9_t11+t9_c11	662.57	6.28	904.90	30.44	585.13	13.65	650.46	16.90	471.87	7.61	699.77	6.15	1144.99	35.13	594.83	29.64	1426.09	32.93	1372.50	33.85
CLA_c11_t13	38.06	10.62	41.59	4.52	31.16	4.43	30.11	3.97	28.19	0.75	37.37	2.27	35.52	2.79	49.55	5.76	32.37	3.09	47.53	7.65
CLA_t10_c12	46.86	6.59	46.69	4.54	0.00	0.00	5.28	0.15	0.00	0.00	0.00	0.00	0.00	0.00	48.58	4.20	0.00	0.00	44.17	4.35
CLA_c8_c10	0.00	0.00	28.80	3.79	39.46	3.88	55.60	3.47	21.84	1.50	35.32	1.28	52.66	4.19	22.35	3.25	80.23	1.32	113.23	3.93
CLA_c9_c11	0.00	0.00	0.00	0.00	2.21	0.44	0.00	0.00	0.00	0.00	2.43	1.18	0.00	0.00	0.00	0.00	0.00	0.00	4.84	1.48
CLA_c10_c12	0.00	0.00	0.00	0.00	2.40	0.12	0.00	0.00	0.00	0.00	0.00	0.00	0.00	0.00	0.00	0.00	0.00	0.00	0.00	0.00
CLA_c11_c13	0.00	0.00	45.57	6.08	0.00	0.00	0.00	0.00	0.00	0.00	0.00	0.00	0.00	0.00	0.00	0.00	0.00	0.00	0.00	0.00
CLA_t11_t13	0.00	0.00	40.97	3.83	24.74	3.92	0.00	0.00	0.00	0.00	0.00	0.00	0.00	0.00	0.00	0.00	21.62	0.80	24.41	7.42
CLA_t12_t14	12.77	2.07	29.75	7.94	39.22	5.00	29.14	3.16	25.53	2.29	25.97	3.10	39.34	4.91	18.11	1.23	49.69	6.21	48.04	6.57
CLA_t9_t11+t10_t12	14.39	1.43	21.42	4.86	16.83	1.08	15.86	1.28	10.45	0.62	15.69	2.34	16.14	1.18	0.00	0.00	29.13	8.25	19.23	6.00
C18:3_c9_t11_t15	0.00	0.00	21.21	3.55	23.00	0.40	29.40	3.08	14.34	4.30	17.76	0.97	28.53	0.86	13.08	1.76	45.95	4.27	52.13	14.76
C20:2	22.58	3.88	21.27	2.24	23.20	4.26	24.26	4.61	21.87	2.33	27.72	4.17	30.51	1.87	18.54	1.58	17.37	14.73	37.29	3.26
CLA_t8_t10	5.02	0.94	5.72	0.73	0.00	0.00	0.00	0.00	0.00	0.00	0.00	0.00	0.00	0.00	0.00	0.00	0.00	0.00	0.00	0.00
CLA_t7_t9	0.00	0.00	58.50	5.50	0.00	0.00	0.00	0.00	0.00	0.00	0.00	0.00	0.00	0.00	0.00	0.00	0.00	0.00	0.00	0.00
C18:3_c9_t11_c15	32.66	0.67	56.58	2.18	46.08	2.53	51.97	3.74	38.51	3.87	43.36	0.91	67.23	3.21	59.85	4.41	87.75	2.67	101.55	1.97
C22:0	47.28	1.76	34.20	5.03	46.21	1.36	52.53	1.65	46.31	2.63	72.92	4.26	59.84	4.46	59.01	3.71	52.20	0.10	87.66	3.95
C20:3n6	27.65	4.03	21.29	2.20	64.70	3.08	59.95	1.78	73.28	3.15	60.85	2.68	76.65	2.78	23.91	0.91	57.13	3.98	56.27	2.05
C18:4_n3	0.00	0.00	17.01	2.46	16.01	0.58	14.08	1.29	12.89	1.49	20.41	3.04	16.18	1.06	7.38	0.75	16.18	0.72	23.40	0.86
C22:1n9	28.47	4.37	14.98	1.09	11.42	0.55	13.80	2.76	10.69	2.69	15.70	2.76	16.93	1.10	14.24	1.89	16.01	1.01	27.53	1.15
C20:3n3	9.11	2.63	122.92	11.98	10.90	5.07	10.48	4.48	13.28	1.60	17.06	0.31	97.85	3.88	10.73	1.06	0.00	0.00	0.00	0.00
C20:4n6	149.98	5.72	125.22	7.33	94.50	3.04	95.48	6.45	132.97	3.77	98.16	7.77	88.84	62.63	154.06	5.93	80.10	2.80	93.18	4.62
C23:0	7.18	5.61	15.97	1.52	20.53	15.41	17.85	18.52	7.09	3.53	35.83	22.12	26.53	21.10	24.88	10.65	27.59	13.70	24.34	30.44
C22:2	15.36	10.92	14.38	0.73	45.22	1.70	40.92	3.29	22.35	1.89	46.84	1.82	52.91	13.18	30.99	2.79	45.66	3.09	41.32	2.42
C20:5n3 EPA	25.58	0.67	75.95	2.07	48.85	1.73	53.05	1.18	39.23	1.53	61.73	2.12	54.01	8.03	48.05	3.73	67.97	2.35	72.23	1.69
C24:0	21.42	0.58	23.05	2.67	42.29	0.61	45.83	0.43	40.77	1.94	55.85	3.51	38.69	21.24	32.21	1.37	46.05	1.01	78.58	1.73
C24:1	12.29	0.89	10.50	1.78	11.24	0.98	10.96	5.74	9.16	5.05	16.86	1.65	10.78	3.29	11.61	1.57	10.98	3.92	14.95	2.55
C22:6n3 DHA	18.88	5.57	41.44	4.12	10.29	5.49	5.15	4.04	20.38	18.71	16.50	2.70	11.42	0.86	35.92	2.55	15.48	1.31	16.28	3.12

**Table S1** supplementary materials. Fatty acid contend in studied cheese [mg $100g^{-1}$ of fat content]	S30: Comte BadozReserva		S31: Gruyeressereserve		S32: ParmiggianoReggiano		S33: ManchegoForlasa		S34: OssauIraty		S35: Bleud'Auvergne		S36: StiltonColston		S37: Fourmed' Ambert		S38: Roquefort Papillon	
	X	SD	X	SD	X	SD	X	SD	X	SD	X	SD	X	SD	X	SD	X	SD
C4:0	3609.23	930.64	3263.57	366.42	3019.70	386.51	3063.43	182.41	3438.99	267.16	3464.69	275.16	3666.80	216.97	3670.70	832.19	3486.28	508.71
C6:0	2223.56	22.47	2231.94	84.13	1938.70	26.83	2578.22	28.31	3008.89	50.89	2248.84	57.02	2330.48	33.14	2181.21	85.26	2922.59	112.50
C8:0	1658.13	23.59	1671.90	56.13	1470.00	24.79	3140.02	42.40	3612.32	76.72	1671.72	62.78	1720.12	25.22	1603.25	61.71	3548.72	151.81
C10:0	2700.97	62.21	2756.93	96.20	2517.62	46.41	7417.40	69.88	8018.42	164.82	2772.27	82.48	2858.79	44.04	2609.70	112.38	8613.78	323.25
C12:0	2834.22	54.81	2868.33	72.22	2744.72	37.44	4193.80	54.07	4060.80	56.50	2910.66	14.16	3216.25	36.90	2744.12	129.19	4801.73	171.60
C13:0_iso	58.36	3.24	51.52	8.53	31.72	3.29	22.55	5.44	34.65	1.52	46.66	3.60	33.61	3.48	51.04	5.44	40.21	3.87
C10:1	87.98	7.73	87.90	2.67	83.66	1.80	54.41	0.62	40.83	2.16	80.11	1.40	95.58	1.63	77.38	6.29	52.69	2.80
C13:0_ai	87.07	5.65	457.77	64.07	78.53	6.57	83.86	5.96	63.41	6.35	78.63	0.95	90.92	6.17	82.77	9.90	88.08	9.57
C14:0_iso	154.30	3.56	116.50	5.19	111.62	1.74	82.83	1.69	123.21	4.16	122.92	3.82	85.04	1.50	126.78	6.12	146.65	5.32
C14:0	9834.34	196.25	9774.61	227.98	8957.82	117.67	9470.44	146.86	9257.64	114.79	9538.15	59.59	9916.19	144.38	9619.09	479.88	11033.99	375.22
C15:O_iso	350.41	7.47	262.65	5.95	199.70	4.75	188.81	7.26	244.67	2.95	285.03	3.97	216.98	5.28	296.18	13.05	300.39	11.39
C15:O_ai	606.44	13.30	508.76	16.94	430.04	8.18	391.99	10.68	423.57	6.89	537.30	8.22	394.21	2.54	531.01	29.31	465.50	13.92
C14:1_c9_(n-5)	801.48	15.43	901.56	18.35	886.48	17.90	217.06	13.50	143.91	5.46	747.43	11.35	907.31	5.48	800.29	40.94	186.46	4.58
C15:0	1096.09	20.98	1047.61	34.72	1066.47	12.28	1035.55	24.96	942.76	9.16	1067.60	12.93	1000.05	7.39	987.07	38.71	1109.83	36.69
C16:0_iso	48.17	3.11	48.99	2.30	66.08	1.37	72.87	4.53	72.86	1.81	49.59	2.66	31.53	2.75	39.44	0.55	87.03	7.16
C16:0_ai	286.65	55.64	245.87	5.68	264.78	5.01	248.42	4.63	259.43	18.95	277.41	5.10	229.88	12.29	303.57	19.95	276.70	13.00
C15:1_n10_(n-5)	18.05	0.92	14.60	10.40	17.12	6.11	17.00	2.72	13.64	1.79	17.73	3.50	17.31	1.19	15.95	1.93	13.44	2.66
C16:0	22301.47	469.44	25016.49	559.54	27481.12	400.31	20796.39	363.49	20202.86	241.18	23279.14	166.87	29151.90	532.86	24253.00	1259.50	23353.64	839.34
C17:O_iso	574.52	13.96	453.13	15.06	326.42	6.52	425.97	28.44	410.29	4.43	510.26	6.51	335.13	12.86	486.65	29.95	383.57	15.21
C17:O_ai	227.16	5.42	208.76	10.61	183.90	4.14	231.03	2.41	242.60	6.37	230.05	19.44	130.68	4.58	202.28	10.86	240.54	7.68
C16:1_t9	433.07	11.89	388.71	10.44	350.94	3.71	388.74	6.55	351.59	7.48	435.45	5.67	346.93	6.49	426.85	20.26	389.22	12.49
C16:1_c9_(n-7)	1062.77	23.56	1270.80	32.44	1455.06	28.38	860.12	18.02	604.85	4.94	1265.41	10.80	1483.58	12.48	1371.40	73.49	731.01	24.70
C16:1_t10	114.55	2.87	103.58	4.38	33.65	4.73	27.41	3.60	46.44	4.46	87.73	2.57	41.76	2.71	86.98	3.66	42.47	0.80
C16:1_t11_+_t12	27.43	2.30	30.55	2.09	35.01	4.66	20.33	3.96	16.67	1.06	35.24	1.77	39.18	4.25	41.05	3.23	21.17	0.38
C17:0	652.62	12.85	634.41	14.81	629.75	5.14	666.19	13.11	608.96	6.65	682.17	3.33	583.57	17.82	650.86	38.95	730.74	27.77
C18:0_iso	66.08	5.56	62.41	2.50	53.66	1.27	67.90	5.36	66.32	7.99	70.52	2.34	49.98	3.54	70.55	5.29	76.03	8.65
C18_0_ai	36.10	2.12	30.01	3.49	37.97	4.44	47.51	1.49	38.67	0.80	32.85	3.53	34.40	3.40	35.11	1.02	40.31	2.36
C17:1_c9	75.00	0.83	60.09	1.29	18.31	1.70	16.16	2.51	26.11	0.58	66.37	0.93	57.17	2.75	58.53	0.98	34.33	1.07
C17:1_c10_(n-7)	216.33	5.62	215.16	4.28	215.85	3.32	250.77	10.65	174.47	2.99	249.60	0.95	186.75	3.49	247.32	12.17	225.92	7.21
C18:0	9057.77	171.77	8300.31	215.36	7313.71	93.07	7938.67	129.42	9273.51	132.43	9197.53	76.92	7910.73	196.56	9312.24	510.55	7132.73	301.89
C18:1_t6	16.81	0.94	18.09	0.81	23.14	2.36	20.91	1.27	22.00	3.24	14.01	10.17	19.46	1.66	21.03	4.35	7.33	3.99
C18:1_t7	20.76	3.05	18.15	2.98	19.96	3.71	30.39	4.01	21.68	1.69	24.29	5.79	20.02	0.52	17.94	2.68	17.11	4.39

**Table S1** supplementary materials. Fatty acid contend in studied cheese [mg 100g^{-1} of fat content]	S30: Comte BadozReserva		S31: Gruyeressereserve		S32: ParmiggianoReggiano		S33: ManchegoForlasa		S34: OssauIraty		S35: Bleud'Auvergne		S36: StiltonColston		S37: Fourmed' Ambert		S38: Roquefort Papillon	
	X	SD	X	SD	X	SD	X	SD	X	SD	X	SD	X	SD	X	SD	X	SD
C18:1_t8	202.39	7.49	179.67	8.71	214.64	4.15	272.23	6.35	198.04	8.59	175.56	8.49	188.00	7.42	177.21	13.33	98.43	2.67
C18:1_t9	234.46	11.46	193.43	3.98	210.56	1.18	241.98	8.39	231.67	8.93	224.16	5.03	213.12	5.73	228.33	13.12	170.02	12.34
C18:1_t10	0.00	0.00	0.00	0.00	326.60	4.70	1446.90	24.86	0.00	0.00	0.00	0.00	278.03	7.72	0.00	0.00	140.89	9.20
C18:1_t11	3311.78	69.93	2249.52	60.20	665.52	15.85	864.97	21.26	1697.21	22.05	2459.86	18.74	898.70	31.94	2000.96	108.61	947.26	99.35
C18:1_t12	205.69	4.14	201.78	3.88	229.52	3.91	294.41	7.65	272.26	4.61	204.53	4.56	256.38	6.69	209.41	15.92	140.11	8.93
C18:1_t13+t14	552.26	11.86	605.79	15.66	560.28	7.20	699.40	12.21	681.29	10.93	565.46	1.96	656.99	16.33	544.12	29.79	361.37	20.64
C18:1_c9_(n-9c)	17332.81	398.85	15410.05	401.87	16759.50	247.77	14917.22	239.85	13987.43	188.91	18407.21	128.89	14744.46	175.83	19561.39	1052.65	12904.21	519.08
C18:1_c10	414.00	15.59	0.00	0.00	0.00	0.00	0.00	0.00	0.00	0.00	0.00	0.00	0.00	0.00	0.00	0.00	26.17	1.27
C18:1_c11	438.48	6.66	372.11	6.65	546.46	10.08	511.20	9.62	353.70	6.24	491.72	3.12	417.08	7.62	524.49	27.53	316.95	16.20
C18:1_c12	106.96	0.76	107.22	7.65	220.49	2.74	280.42	6.21	189.89	9.19	113.33	3.02	171.06	5.75	141.08	13.43	120.30	7.21
C18:1_c13	63.57	3.14	64.17	2.16	126.93	87.11	59.12	2.17	58.38	4.34	77.41	4.70	67.10	2.39	79.70	8.21	44.21	5.58
C18:1_c14	293.59	7.84	281.09	5.87	233.59	3.70	275.29	5.12	351.85	14.90	299.47	4.97	287.23	5.72	285.23	18.37	211.69	12.76
C18:1_c15	78.44	4.94	72.27	4.02	137.79	9.04	48.34	4.80	100.38	46.48	88.25	2.40	62.63	29.49	85.92	13.21	168.65	36.38
C18:1_c16	109.32	4.60	101.18	6.22	2.71	0.37	0.00	0.00	110.52	1.50	95.81	7.92	76.83	6.93	88.75	9.07	67.32	1.36
C18:2 _t7_c9	93.02	5.61	60.50	1.60	26.76	9.97	60.15	3.73	37.39	11.76	64.31	3.92	40.26	9.43	53.47	14.59	24.34	0.78
C18:2 _c9_t11	90.13	10.16	57.27	3.47	18.38	1.05	9.78	0.68	43.00	7.76	66.24	1.93	21.38	3.86	51.44	4.65	23.56	1.38
C18:2_t10_c12	19.20	2.60	16.25	1.45	0.00	0.00	21.18	1.22	18.44	4.39	18.56	1.21	73.93	96.98	17.29	1.39	14.54	1.33
C18:2n6t (t9_t12)	220.85	7.28	203.46	2.04	169.10	0.57	265.82	3.41	253.44	2.67	205.20	4.21	133.65	98.29	198.28	12.60	157.67	11.79
C18:2_c9_t13	10.15	4.93	11.83	0.85	19.31	2.92	2.55	1.14	5.83	1.04	15.79	2.97	36.52	37.49	12.20	3.59	0.00	0.00
C18:2_t9_c13	99.57	7.97	78.07	5.08	113.55	2.57	68.15	7.67	85.67	10.51	115.14	2.59	90.58	1.91	135.69	14.67	72.14	4.01
C18:2_t8_c12	90.67	6.06	86.59	0.54	77.31	2.28	107.02	7.36	115.40	8.14	86.72	2.24	87.04	6.42	92.64	5.91	78.23	6.34
C18:2_t8_c13	88.06	3.31	73.65	3.34	76.44	5.20	96.60	0.52	107.71	5.92	83.32	3.13	65.15	35.33	84.91	8.38	68.49	6.26
C18:2_c9_t12	70.53	2.51	47.27	3.97	24.41	2.90	21.85	3.80	33.97	1.13	52.23	3.24	53.90	50.91	47.51	6.74	22.66	5.65
C18:2_t9_c12	398.98	13.77	284.34	2.55	57.84	3.43	115.33	15.92	232.12	4.01	281.28	3.33	90.84	60.73	243.53	11.62	154.76	13.72
C18:2n6c (c9_c12)	1200.64	34.76	1234.31	26.06	1920.09	29.13	2466.98	29.17	1793.25	31.37	1357.41	12.60	1454.81	30.22	1417.96	68.96	1735.74	76.22
C18:2_t11_c15	29.47	3.86	0.00	0.00	0.00	0.00	2.65	0.38	0.00	0.00	0.00	0.00	0.00	0.00	0.00	0.00	0.00	0.00
C18:2_c9_c15	26.55	13.97	39.08	2.34	41.44	5.29	10.39	0.62	29.99	7.49	39.89	0.81	37.90	7.03	41.74	5.60	17.84	9.11
C18:3_c6_c9_c12	60.58	66.13	20.90	5.45	0.00	0.00	0.00	0.00	20.53	3.92	20.90	4.38	22.09	5.30	18.99	3.29	14.10	0.46
C20:0+C18:3_t9_t12_t15	99.93	65.78	136.19	5.32	132.43	2.40	236.66	2.64	233.09	1.61	155.66	3.42	171.47	10.89	146.64	8.71	277.75	14.81
C18:3_t9_t12_c15	0.00	0.00	0.00	0.00	0.00	0.00	20.20	4.99	18.59	1.99	28.58	2.35	0.00	0.00	0.00	0.00	0.00	0.00
C18:3_t9_c12_t15	26.71	1.79	0.00	0.00	0.00	0.00	0.00	0.00	0.00	0.00	0.00	0.00	0.00	0.00	11.72	3.23	0.00	0.00
C18:3n6 (c6_c9_c12)	23.02	1.18	26.33	3.83	37.40	4.55	63.56	2.00	45.83	3.66	26.34	4.72	28.02	5.22	27.81	3.87	35.39	4.87

**Table S1** supplementary materials. Fatty acid contend in studied cheese [mg 100g-1 of fat content]	S30: Comte BadozReserva		S31: Gruyeressereserve		S32: ParmiggianoReggiano		S33: ManchegoForlasa		S34: OssauIraty		S35: Bleud'Auvergne		S36: StiltonColston		S37: Fourmed' Ambert		S38: Roquefort Papillon	
	X	SD	X	SD	X	SD	X	SD	X	SD	X	SD	X	SD	X	SD	X	SD
C18:3_c9_t12_t15+c9_c12_t15	13.86	1.30	0.00	0.00	0.00	0.00	0.00	0.00	0.00	0.00	0.00	0.00	5.33	1.26	3.33	0.22	0.00	0.00
C18:3_c9_t12_c15	18.30	1.13	16.41	1.46	120.69	7.24	0.00	0.00	0.00	0.00	118.34	7.67	171.05	31.05	123.55	14.34	13.60	0.71
C18:3_t9_c12_c15	103.69	56.31	119.86	9.63	96.29	37.94	0.00	0.00	0.00	0.00	127.88	0.88	102.96	64.62	133.93	10.22	0.00	0.00
C20:1	249.66	363.30	35.87	1.96	48.01	3.10	48.60	1.10	35.33	1.82	46.25	3.46	49.22	2.19	50.91	2.30	40.59	5.06
C18:3n3 (c9_c12_c15)	737.34	31.13	831.35	28.98	459.16	5.76	399.37	5.31	722.94	10.78	702.13	10.50	361.10	4.21	596.86	27.35	903.72	45.17
CLA_c9_t11+t9_c11	1664.84	35.68	1000.66	27.09	408.09	10.49	541.49	15.67	726.37	16.74	1106.84	10.92	471.90	14.95	916.52	53.70	494.06	26.44
CLA_c11_t13	36.29	4.25	31.91	1.89	38.26	6.59	78.17	3.36	81.80	10.88	36.02	1.92	34.57	2.00	33.84	4.57	93.03	6.72
CLA_t10_c12	87.75	7.70	0.00	0.00	0.00	0.00	0.00	0.00	0.00	0.00	0.00	0.00	0.00	0.00	5.68	1.39	97.60	4.65
CLA_c8_c10	77.54	3.48	46.76	2.62	0.00	0.00	0.00	0.00	37.62	1.83	55.93	1.33	26.40	2.70	45.98	6.27	21.86	1.37
CLA_c9_c11	2.88	0.22	0.00	0.00	0.00	0.00	0.00	0.00	2.81	0.12	5.17	0.20	0.00	0.00	0.00	0.00	0.00	0.00
CLA_c10_c12	0.00	0.00	0.00	0.00	0.00	0.00	0.00	0.00	0.00	0.00	0.00	0.00	0.00	0.00	0.00	0.00	0.00	0.00
CLA_c11_c13	0.00	0.00	0.00	0.00	0.00	0.00	0.00	0.00	0.00	0.00	0.00	0.00	0.00	0.00	0.00	0.00	0.00	0.00
CLA_t11_t13	29.40	3.21	10.56	0.28	0.00	0.00	0.00	0.00	0.00	0.00	0.00	0.00	0.00	0.00	0.00	0.00	0.00	0.00
CLA_t12_t14	47.25	4.08	45.42	4.84	23.95	4.16	29.38	10.04	41.46	4.46	37.58	1.24	29.36	1.94	38.32	1.39	21.10	2.02
CLA_t9_t11+t10_t12	19.77	3.38	20.35	2.94	17.19	1.83	15.08	3.06	24.13	7.41	17.42	3.29	16.77	4.09	16.12	2.76	31.23	3.65
C18:3_c9_t11_t15	51.99	9.17	35.21	4.41	23.35	3.15	0.00	0.00	25.93	7.05	29.91	2.19	22.88	8.73	28.14	1.42	19.06	3.34
C20:2	25.62	5.82	26.62	0.74	33.15	0.30	26.04	2.01	32.93	2.05	23.72	1.69	24.77	2.78	21.51	3.81	25.26	4.73
CLA_t8_t10	0.00	0.00	0.00	0.00	0.00	0.00	0.00	0.00	0.00	0.00	0.00	0.00	0.00	0.00	0.00	0.00	0.00	0.00
CLA_t7_t9	0.00	0.00	0.00	0.00	0.00	0.00	0.00	0.00	0.00	0.00	0.00	0.00	0.00	0.00	0.00	0.00	0.00	0.00
C18:3_c9_t11_c15	92.21	6.02	68.29	2.73	24.05	3.06	52.42	3.43	106.62	2.91	70.94	3.73	33.19	0.61	55.09	3.13	73.00	7.17
C22:0	64.34	5.62	58.32	4.04	54.70	1.79	96.49	4.11	78.76	47.03	68.57	3.94	63.05	2.02	63.11	2.12	136.18	9.38
C20:3n6	58.75	1.79	63.63	2.01	109.19	3.27	39.53	3.50	28.85	0.83	61.51	4.58	76.13	1.96	67.16	2.03	24.07	2.44
C18:4_n3	19.53	0.37	16.33	2.17	19.70	4.03	9.97	2.75	16.90	3.74	14.19	8.62	18.71	1.66	15.36	1.07	0.00	0.00
C22:1n9	21.22	2.31	19.98	0.56	12.93	4.84	13.78	1.61	11.90	0.94	17.67	2.38	27.96	16.30	14.14	2.47	16.46	3.34
C20:3n3	22.68	1.13	21.47	2.52	10.84	1.40	0.00	0.00	126.15	11.70	17.21	1.71	17.81	4.08	18.63	3.24	18.50	4.41
C20:4n6	89.38	3.28	95.23	4.06	167.65	5.36	245.24	7.94	122.76	38.82	102.09	5.19	112.53	4.98	118.50	11.11	147.13	9.90
C23:0	39.09	20.26	33.11	27.28	16.31	15.08	6.93	3.64	7.74	4.38	20.04	13.12	26.54	15.12	33.75	22.72	33.03	42.50
C22:2	64.28	5.00	45.21	1.60	15.23	1.58	18.18	0.89	53.58	4.99	56.07	6.25	44.21	2.26	47.24	2.96	69.96	5.53
C20:5n3 EPA	71.40	1.33	75.12	4.01	40.05	5.61	39.58	1.94	59.65	7.23	69.96	1.63	46.68	2.81	57.02	2.29	68.89	3.51
C24:0	56.17	3.53	58.59	5.17	46.41	1.65	61.51	24.99	76.84	31.19	65.70	6.83	56.95	15.68	58.68	4.53	74.73	9.25
C24:1	12.06	1.68	9.15	4.40	6.62	2.19	14.18	2.29	17.04	7.74	14.96	2.42	15.20	0.41	10.57	6.00	26.52	1.31
C22:6n3 DHA	15.58	2.12	14.87	1.71	70.69	48.89	35.68	1.33	45.68	2.84	13.19	1.45	21.46	9.65	12.32	3.10	51.26	2.62

**Table S2** supplementary materials. Fatty acid contend in studied cheese [mg $100g^{-1}$ of cheese]	S1: Camembert l'aromatique		S2: Camembert de Caractere. Roi du Chateau		S3: BucheFondante		S4: Gorgonzola Cremoso		S5: Gorgonzola Intense		S6: Cow mountain cheese non smoked		S7: Cow mountain cheese smoked		S8: Sheep mountain cheese "oscypek"non smoked		S9: Sheep mountain cheese "oscypek" smoked		S10: L'amidu Chambertin	
	x	SD	X	SD	X	SD	X	SD	X	SD	X	SD	X	SD	X	SD	X	SD	X	SD
C4:0	571.1	21.9	1149.2	83.6	948.6	78.2	407.8	21.3	981.9	130.4	945.1	47.3	1138.1	152.4	909.1	78.6	1245.2	161.6	720.5	86.5
C6:0	369.2	15.7	671.5	11.8	509.0	19.2	341.8	6.3	585.2	12.4	471.6	2.1	540.1	3.3	581.8	42.1	685.4	8.1	462.2	27.9
C8:0	270.2	8.4	491.5	7.6	700.0	29.8	260.3	2.5	437.3	10.4	323.4	5.3	376.2	5.7	593.1	52.9	719.7	8.4	340.2	20.0
C10:0	456.9	12.4	805.4	13.3	1757.5	78.7	472.9	4.7	749.3	19.0	483.8	9.6	578.8	12.2	1191.9	118.8	1452.6	34.7	633.3	39.3
C12:0	480.4	15.4	846.3	11.2	766.2	37.0	523.2	5.1	799.2	16.1	490.5	7.1	595.8	6.0	721.1	45.0	839.5	14.3	692.7	48.1
C13:0_iso	7.8	0.4	11.9	1.4	6.1	0.5	5.3	0.3	8.4	0.3	11.1	0.4	12.8	0.9	13.4	3.3	11.8	1.0	8.1	0.8
C10:1	13.2	1.0	24.8	0.6	10.1	0.5	13.8	0.7	21.8	0.6	11.3	0.5	11.4	6.6	13.8	1.2	15.4	2.3	18.0	1.1
C13:0_ai	12.5	0.8	24.5	1.6	16.9	0.5	15.1	2.3	21.7	0.4	11.5	0.4	14.5	1.4	15.5	2.2	17.6	1.4	18.2	0.7
C14:0_iso	18.7	0.5	30.2	0.6	17.3	0.9	13.9	0.3	22.1	0.2	33.1	0.3	30.7	0.0	36.1	3.3	36.5	0.5	24.7	1.8
C14:0	1616.5	55.2	2728.9	26.8	1795.1	86.2	1713.9	19.9	2531.4	42.6	1831.8	21.0	2087.1	25.3	2130.6	142.1	2290.8	19.7	2218.2	155.2
C15:O_iso	43.4	2.0	65.4	0.7	39.3	2.7	32.4	0.3	49.2	1.3	75.9	1.2	87.4	1.5	91.0	8.9	90.6	3.1	51.5	3.4
C15:O_ai	81.3	2.3	134.3	1.0	61.1	2.7	65.3	1.3	102.6	2.1	117.6	1.1	125.1	1.9	163.7	13.6	170.4	0.5	113.5	7.5
C14:1_c9_(n-5)	122.5	4.5	239.9	2.1	29.1	2.1	135.5	2.1	218.7	3.8	133.2	1.6	158.2	3.2	72.6	5.4	77.1	1.1	151.6	11.1
C15:0	167.6	4.8	294.5	1.7	170.1	11.2	185.2	2.3	264.9	3.0	248.9	3.3	256.9	6.3	293.7	21.7	309.2	4.1	234.8	18.3
C16:0_iso	6.3	0.6	11.0	1.8	11.0	1.4	15.6	19.0	7.6	0.6	13.2	0.3	17.7	0.4	19.5	1.9	18.4	0.5	9.1	0.6
C16:0_ai	51.4	1.9	76.9	3.2	47.5	4.9	40.8	4.7	59.2	0.5	70.2	1.9	70.2	0.3	73.9	6.5	75.7	0.2	60.4	2.4
C15:1_n10_(n-5)	2.7	0.1	4.3	2.8	2.9	0.4	3.7	1.1	5.2	1.5	3.8	0.3	4.4	0.4	4.0	0.2	4.7	0.2	3.4	0.5
C16:0	4026.9	140.2	7443.8	63.1	4557.0	231.7	4839.7	64.1	6986.5	113.6	5293.1	60.4	5638.2	70.2	4892.9	374.7	5115.1	7.5	5355.4	368.6
C17:O_iso	72.7	2.9	108.4	1.3	80.2	3.9	58.5	0.9	86.7	2.5	116.2	2.0	129.4	2.3	192.9	10.1	207.5	0.6	84.4	5.7
C17:O_ai	35.2	1.7	59.5	1.6	47.8	2.2	23.4	0.2	39.1	1.5	55.1	1.3	65.0	1.7	84.3	7.4	83.8	1.7	34.5	2.4
C16:1_t9	68.8	1.9	118.1	0.2	69.6	2.3	63.1	1.6	93.3	1.5	97.3	0.5	100.8	3.4	115.4	12.9	113.3	0.5	95.9	7.7
C16:1_c9_(n-7)	180.1	8.5	400.9	5.5	103.6	6.8	208.5	2.1	346.1	7.3	301.0	4.3	329.6	4.5	193.3	20.3	193.8	3.2	228.9	15.9
C16:1_t10	11.8	0.8	16.5	1.7	5.5	0.7	4.6	0.3	8.1	0.7	26.1	0.4	32.1	1.0	21.0	2.3	0.0	0.0	8.6	0.7
C16:1_t11_+_t12	8.1	2.8	15.2	1.2	3.1	0.3	6.0	0.3	8.6	2.5	9.5	0.2	9.3	0.4	10.0	9.8	20.6	0.3	6.8	1.6
C17:0	103.6	3.6	185.1	2.2	126.7	6.5	111.0	1.1	158.7	4.5	177.1	1.9	174.7	2.5	178.7	19.0	176.9	1.4	135.3	9.2
C18:0_iso	10.1	0.8	18.1	0.4	8.9	0.4	7.7	2.3	12.4	0.5	19.9	1.0	19.9	0.4	19.3	2.1	20.0	1.2	11.8	0.8
C18_0_ai	5.7	0.0	10.8	1.4	8.8	1.2	13.7	14.9	8.4	0.8	8.1	0.5	9.3	0.2	10.1	1.9	8.3	3.2	7.0	1.2
C17:1_c9	7.2	0.1	11.5	1.1	5.5	0.5	2.8	0.6	4.0	0.8	15.3	0.5	15.8	0.6	18.0	2.3	16.1	1.6	6.0	0.6
C17:1_c10_(n-7)	28.9	0.6	65.7	0.5	35.6	1.2	32.0	1.8	50.9	2.5	69.5	0.9	67.3	1.5	60.0	6.3	56.5	1.4	36.3	2.2
C18:0	1665.8	58.9	2589.8	16.1	1778.0	96.5	1606.9	19.2	2337.5	40.4	2458.8	32.9	2750.0	41.9	2490.9	198.8	2524.4	45.1	1761.3	121.2
C18:1_t6	3.4	0.2	6.1	0.6	3.6	0.0	3.9	0.1	5.7	0.3	4.1	0.5	4.5	0.7	3.0	2.1	3.1	2.8	4.4	0.3
C18:1_t7	3.3	0.8	6.8	0.8	5.3	1.6	4.2	1.4	6.0	0.5	3.8	0.5	5.7	1.6	6.1	1.4	5.7	1.7	4.8	0.6

**Table S2** supplementary materials. Fatty acid contend in studied cheese [mg 100g^{-1} of cheese]	S1: Camembert l'aromatique		S2: Camembert de Caractere. Roi du Chateau		S3: BucheFondante		S4: Gorgonzola Cremoso		S5: Gorgonzola Intense		S6: Cow mountain cheese non smoked		S7: Cow mountain cheese smoked		S8: Sheep mountain cheese "oscypek"non smoked		S9: Sheep mountain cheese "oscypek" smoked		S10: L'amidu Chambertin	
	x	SD	X	SD	X	SD	X	SD	X	SD	X	SD	X	SD	X	SD	X	SD	X	SD
C18:1_t8	33.8	1.8	52.6	3.8	44.5	2.3	43.4	1.9	64.7	0.2	37.6	1.4	39.7	0.8	58.3	2.1	62.2	0.3	46.8	3.2
C18:1_t9	36.8	1.6	60.5	1.9	53.9	3.5	43.1	0.9	68.6	1.2	44.2	0.5	49.0	2.4	72.9	2.3	79.7	4.0	49.2	4.3
C18:1_t10	0.0	0.0	60.7	7.3	71.5	3.6	105.4	3.3	154.8	5.7	0.0	0.0	0.0	0.0	0.0	0.0	0.0	0.0	0.0	0.0
C18:1_t11	345.3	14.1	391.3	36.6	197.4	10.1	149.5	1.2	223.7	4.6	540.1	7.8	596.1	10.9	1059.2	97.0	1250.5	23.8	386.8	26.1
C18:1_t12	43.2	1.7	67.0	1.7	53.5	3.7	61.8	0.9	92.8	0.9	40.7	0.6	53.9	0.8	87.7	4.1	95.6	3.2	55.6	4.5
C18:1_t13+t14	115.1	4.2	168.9	0.7	107.1	4.9	136.4	2.3	202.1	4.2	124.2	1.7	176.5	8.2	247.6	13.1	262.7	5.1	133.6	9.6
C18:1_c9_(n-9c)	2454.6	93.2	4822.0	39.0	3333.1	157.6	2602.6	30.3	4327.7	74.0	4311.8	55.8	4514.1	70.7	4211.1	332.9	4304.1	57.1	3101.2	209.7
C18:1_c10	0.0	0.0	0.0	0.0	0.0	0.0	70.3	11.6	0.0	0.0	0.0	0.0	0.0	0.0	0.0	0.0	0.0	0.0	0.0	0.0
C18:1_c11	79.2	1.9	138.1	1.4	82.3	8.0	90.5	8.7	136.5	3.4	116.8	0.7	115.3	2.5	85.2	5.2	89.2	1.4	93.4	6.9
C18:1_c12	29.4	0.9	53.3	0.7	43.3	2.7	62.6	1.1	99.4	3.3	21.9	0.3	28.7	1.0	34.0	5.0	31.5	0.7	44.7	3.1
C18:1_c13	12.2	0.5	23.5	0.9	11.2	2.1	13.7	0.1	22.4	0.9	35.5	30.2	19.2	1.7	21.6	0.7	23.9	0.1	13.5	0.3
C18:1_c14	56.6	1.5	80.0	1.2	56.2	7.1	61.5	1.2	82.0	2.6	50.7	34.0	98.6	1.6	130.8	8.0	136.4	0.4	61.8	3.0
C18:1_c15	14.4	0.8	21.8	9.0	30.4	9.9	13.3	0.8	18.9	0.4	25.4	0.4	26.3	1.5	37.3	3.9	23.5	18.1	9.9	6.5
C18:1_c16	17.1	0.6	18.2	0.6	15.7	2.1	15.9	0.4	24.1	2.3	21.7	0.8	30.2	0.9	44.8	3.7	51.8	7.5	16.6	1.7
C18:2 _t7_c9	10.0	0.3	12.1	1.7	4.8	0.9	8.5	3.3	10.1	2.4	13.5	5.1	10.9	0.8	18.0	0.2	17.3	3.3	10.8	1.7
C18:2 _c9_t11	9.7	0.6	8.8	0.9	3.8	1.0	3.0	0.5	4.8	0.7	6.3	8.0	19.0	1.3	30.8	1.7	31.1	2.8	8.0	0.6
C18:2_t10_c12	3.2	0.5	5.6	0.8	5.0	1.2	13.9	18.3	5.2	0.4	39.5	0.3	49.6	5.0	8.0	1.5	6.4	1.5	3.5	1.5
C18:2n6t (t9_t12)	30.4	1.0	52.8	7.1	47.7	2.0	34.3	1.1	56.5	1.3	0.0	0.0	58.1	5.7	123.6	8.1	131.5	1.4	39.6	2.0
C18:2_c9_t13	2.7	0.8	20.3	26.7	0.0	0.0	3.1	0.2	5.0	0.5	2.0	0.1	3.0	1.2	0.0	0.0	0.0	0.0	4.1	0.4
C18:2_t9_c13	21.2	1.0	36.3	1.2	11.9	2.2	23.1	2.5	35.9	2.7	20.0	1.0	20.7	1.8	20.7	3.3	19.3	0.1	31.2	2.3
C18:2_t8_c12	12.1	0.3	21.8	1.4	21.4	1.4	14.7	0.8	24.3	0.6	18.3	0.5	24.9	1.4	50.1	3.3	53.3	1.6	17.4	1.9
C18:2_t8_c13	15.0	0.7	21.7	1.0	22.3	7.7	15.0	1.2	24.1	0.9	18.6	1.2	25.5	2.3	36.7	3.1	40.0	0.2	17.6	1.1
C18:2_c9_t12	7.4	0.8	9.4	0.6	4.6	0.6	7.5	6.6	7.5	0.5	34.0	39.9	12.2	0.4	24.0	2.5	29.4	0.1	8.6	0.2
C18:2_t9_c12	42.1	0.9	43.6	2.1	25.8	1.6	9.6	0.8	19.3	1.0	77.4	3.7	98.0	0.7	137.4	9.1	143.7	2.6	38.3	2.7
C18:2n6c (c9_c12)	188.4	5.7	393.0	4.7	469.7	26.8	342.0	5.7	562.6	11.2	321.1	2.5	355.7	8.3	333.8	52.3	305.1	0.3	320.7	18.9
C18:2_t11_c15	0.0	0.0	0.0	0.0	0.0	0.0	4.3	0.5	0.0	0.0	0.0	0.0	0.0	0.0	0.0	0.0	0.0	0.0	0.9	0.1
C18:2_c9_c15	5.9	0.4	11.3	0.2	6.2	0.3	3.7	2.5	10.6	0.2	8.4	0.5	11.5	1.0	11.9	2.4	11.4	1.0	5.2	3.1
C18:3_c6_c9_c12	3.7	0.1	4.6	1.1	3.5	0.5	9.4	11.6	4.6	0.4	4.5	0.9	6.2	0.5	9.1	1.7	9.3	1.0	4.3	0.4
C20:0+C18:3_t9_t12_t15	25.5	0.8	43.5	1.0	45.7	2.7	15.4	11.2	32.2	0.7	45.3	0.2	44.5	1.9	62.9	11.3	54.7	2.6	30.7	1.5
C18:3_t9_t12_c15	0.0	0.0	0.0	0.0	0.0	0.0	4.5	0.4	0.0	0.0	0.0	0.0	0.0	0.0	6.1	1.7	0.0	0.0	0.0	0.0
C18:3_t9_c12_t15	0.0	0.0	0.0	0.0	0.0	0.0	0.0	0.0	0.0	0.0	0.0	0.0	0.0	0.0	0.0	0.0	0.0	0.0	0.0	0.0
C18:3n6 (c6_c9_c12)	4.1	0.2	7.1	0.9	5.3	0.6	4.7	0.4	9.0	0.6	5.1	0.5	7.4	0.7	6.5	1.1	4.8	0.7	6.1	0.5

**Table S2** supplementary materials. Fatty acid contend in studied cheese [mg 100g^{-1} of cheese]	S1: Camembert l'aromatique		S2: Camembert de Caractere. Roi du Chateau		S3: BucheFondante		S4: Gorgonzola Cremoso		S5: Gorgonzola Intense		S6: Cow mountain cheese non smoked		S7: Cow mountain cheese smoked		S8: Sheep mountain cheese “oscypek”non smoked		S9: Sheep mountain cheese “oscypek” smoked		S10: L'amidu Chambertin	
	x	SD	X	SD	X	SD	X	SD	X	SD	X	SD	X	SD	X	SD	X	SD	X	SD
C18:3_c9_t12_t15+c9_c12_t15	0.0	0.0	0.0	0.0	0.0	0.0	0.0	0.0	2.0	0.4	3.0	0.3	6.9	0.8	6.6	2.1	4.5	0.3	3.2	0.3
C18:3_c9_t12_c15	1.8	0.1	6.3	1.2	5.4	0.2	6.5	0.9	0.0	0.0	31.5	0.6	32.2	1.6	6.3	2.2	7.3	4.0	22.7	2.9
C18:3_t9_c12_c15	17.3	0.7	37.2	1.5	0.0	0.0	17.0	0.6	24.1	1.5	10.7	0.3	10.1	1.2	7.0	1.3	7.6	3.5	25.8	3.3
C20:1	7.5	0.5	13.9	0.8	12.1	1.5	21.7	25.9	10.9	1.1	10.1	0.5	1.0	0.2	7.1	0.9	7.7	2.1	9.2	0.6
C18:3n3 (c9_c12_c15)	72.6	2.3	118.2	0.5	110.9	6.4	56.7	2.3	92.6	1.5	207.7	2.1	263.1	4.7	320.5	42.8	299.6	8.5	88.2	5.5
CLA_c9_t11+t9_c11	116.7	4.5	155.9	2.5	121.5	4.8	73.8	1.2	119.2	1.9	196.2	5.1	222.1	6.0	556.4	57.6	662.9	14.3	157.5	10.5
CLA_c11_t13	0.8	0.0	0.0	0.0	10.6	0.8	5.0	0.2	7.0	1.2	13.1	0.9	12.6	1.2	28.0	2.7	0.0	0.0	6.9	0.7
CLA_t10_c12	5.8	0.9	9.2	1.2	0.0	0.0	0.0	0.0	7.4	0.6	0.0	0.0	0.0	0.0	24.0	0.7	19.7	0.4	8.4	0.5
CLA_c8_c10	7.4	1.0	6.5	0.8	0.0	0.0	0.0	0.0	0.0	0.0	16.4	1.2	19.2	0.9	27.2	2.2	14.7	19.9	6.4	0.8
CLA_c9_c11	6.6	0.3	6.9	1.4	0.0	0.0	0.0	0.0	0.0	0.0	1.9	0.6	1.2	0.2	0.0	0.0	30.5	5.5	0.0	0.0
CLA_c10_c12	0.4	0.0	0.0	0.0	0.0	0.0	0.0	0.0	0.0	0.0	0.0	0.0	0.0	0.0	0.0	0.0	1.2	0.1	0.0	0.0
CLA_c11_c13	0.0	0.0	0.0	0.0	0.0	0.0	0.0	0.0	0.0	0.0	0.0	0.0	0.0	0.0	0.0	0.0	0.0	0.0	0.0	0.0
CLA_t11_t13	3.5	0.2	0.0	0.0	0.0	0.0	5.7	0.9	0.0	0.0	4.6	0.4	6.8	0.9	11.8	0.3	10.6	1.1	0.0	0.0
CLA_t12_t14	8.9	0.5	9.4	1.6	3.9	0.2	4.5	1.6	7.9	0.6	10.6	1.3	12.0	0.5	23.7	1.0	23.8	0.0	5.2	0.2
CLA_t9_t11+t10_t12	3.5	0.4	10.4	1.0	3.0	0.5	1.6	0.4	3.4	0.3	3.2	0.8	4.8	0.8	7.9	0.8	7.9	0.5	2.9	0.4
C18:3_c9_t11_t15	3.9	0.1	4.4	0.6	0.0	0.0	3.5	0.9	0.0	0.0	6.8	1.0	7.2	0.8	16.5	1.3	18.7	1.8	3.9	1.0
C20:2	5.1	0.8	7.7	1.7	4.9	1.3	3.0	1.7	7.6	0.6	5.8	0.8	6.0	0.2	7.6	0.6	6.8	0.1	6.8	1.0
CLA_t8_t10	0.0	0.0	0.0	0.0	0.0	0.0	0.0	0.0	0.0	0.0	0.0	0.0	0.0	0.0	0.0	0.0	4.2	1.4	0.0	0.0
CLA_t7_t9	0.0	0.0	0.0	0.0	0.0	0.0	3.8	0.3	0.0	0.0	0.0	0.0	0.0	0.0	0.0	0.0	0.0	0.0	0.0	0.0
C18:3_c9_t11_c15	7.9	0.2	10.5	0.5	10.8	1.6	5.3	2.5	6.4	0.7	10.8	0.3	8.7	6.8	44.6	2.2	50.8	2.2	9.7	0.6
C22:0	9.6	0.4	15.5	0.2	12.9	1.3	7.6	0.9	10.1	0.8	21.3	0.6	20.3	1.0	37.5	5.0	29.3	3.5	11.5	0.9
C20:3n6	11.5	0.4	21.5	0.8	6.3	1.2	16.9	0.1	26.6	1.8	13.8	0.4	16.0	1.0	6.6	0.5	7.2	0.1	19.2	1.9
C18:4_n3	2.4	0.2	4.8	0.5	0.0	0.0	2.1	1.2	3.0	0.2	3.8	0.2	5.2	0.3	2.4	0.3	2.3	0.7	3.8	0.5
C22:1n9	2.1	0.3	3.3	0.9	6.3	0.8	0.0	0.0	1.7	0.2	4.9	0.2	5.6	0.6	4.9	0.7	4.4	0.1	2.6	0.2
C20:3n3	2.7	0.2	3.9	0.7	3.3	0.3	23.0	2.5	0.0	0.0	1.0	0.1	0.0	0.0	2.5	0.8	6.0	1.2	2.6	0.1
C20:4n6	19.8	0.8	36.5	0.3	32.5	1.2	21.7	14.2	46.4	0.3	21.1	0.8	19.6	0.4	23.3	2.9	22.7	0.2	30.9	1.9
C23:0	3.3	3.5	8.9	5.5	5.2	1.0	2.2	1.1	4.5	0.2	2.9	0.6	8.4	9.1	24.6	3.6	12.4	14.1	8.9	0.5
C22:2	7.4	0.6	9.6	0.7	5.5	0.1	2.0	0.1	2.9	0.4	9.3	0.3	10.9	0.1	30.6	1.5	31.9	4.7	5.8	0.4
C20:5n3 EPA	8.4	0.2	12.9	0.2	33.0	2.0	5.0	0.4	9.0	2.9	19.1	0.7	33.8	17.2	23.2	3.5	21.8	0.9	8.3	0.3
C24:0	8.7	0.4	14.7	0.8	30.7	3.0	5.0	0.4	11.2	5.6	19.1	4.3	23.8	8.6	26.6	4.4	20.6	4.5	10.5	0.5
C24:1	1.3	0.7	10.0	13.1	2.6	0.8	2.6	2.1	3.8	3.7	2.9	0.2	3.6	0.5	4.5	2.3	16.0	14.5	8.7	10.9
C22:6n3 DHA	6.7	8.6	4.3	0.8	6.5	0.4	17.2	11.6	19.0	1.4	4.8	0.0	11.1	11.4	20.1	1.9	16.4	1.4	7.4	9.2

**Table S2** supplementary materials. Fatty acid contend in studied cheese [mg $100g^{-1}$ of cheese]	S11: Brie de Meaux		S12: Camembert de Normandie		S13: Epoisses de Bourgogne		S14: Petit Brillat Savarin		S15: Pon'tl'evegue		S16: Fromage de chevre au lait cru (Chevre Ronde)		S17: Crottin de Chavignol		S18: Le Fleuret		S19: Picodon Carte Noire		S20: Sainte Maure de Touraine. Hardy Affineur	
	X	SD	X	SD	X	SD	X	SD	X	SD	X	SD	X	SD	X	SD	X	SD	X	SD
C4:0	605.9	72.5	803.4	98.4	769.7	204.1	968.8	299.6	837.8	197.6	1110.6	100.7	694.8	188.0	509.8	134.9	469.7	87.9	596.4	106.7
C6:0	382.8	12.6	429.7	38.4	476.8	26.9	623.1	13.2	512.3	21.5	771.3	26.5	643.9	4.7	493.5	8.4	458.4	9.1	518.5	13.3
C8:0	292.2	10.8	323.4	28.5	354.0	18.2	459.0	13.4	382.2	14.4	1063.6	44.3	868.2	12.6	651.3	15.7	609.7	8.3	682.0	16.5
C10:0	498.0	17.2	544.8	40.1	622.5	31.7	803.1	21.1	643.1	22.1	2586.7	112.9	2044.8	32.4	1543.8	35.8	1530.6	23.5	1688.0	27.2
C12:0	543.3	17.6	572.7	38.8	669.6	36.6	870.2	15.1	683.0	25.9	1117.0	32.0	866.6	12.9	565.5	14.4	676.3	13.8	744.9	6.5
C13:0_iso	6.1	0.6	10.1	0.8	7.1	0.1	9.1	1.2	11.8	0.3	11.7	0.9	7.9	0.7	5.1	0.6	6.3	0.5	6.4	0.5
C10:1	16.8	1.4	14.8	1.1	18.0	0.7	25.0	0.4	18.4	0.6	13.9	2.2	6.9	1.0	6.0	0.8	6.6	0.3	10.6	0.6
C13:0_ai	15.3	0.2	15.7	2.3	18.7	1.1	22.8	2.1	20.4	1.7	22.7	1.3	15.5	1.1	11.5	0.7	13.1	1.2	18.1	1.0
C14:0_iso	14.5	0.2	25.0	1.8	20.0	1.4	25.2	1.3	32.3	1.0	29.9	0.6	24.4	0.4	13.3	0.5	23.3	0.3	15.2	0.4
C14:0	1761.9	53.8	1818.8	113.4	2155.8	119.6	2809.8	43.2	2261.8	88.4	2546.1	51.4	2044.4	16.1	1391.1	35.8	1738.8	41.6	1853.3	12.0
C15:O_iso	38.5	1.6	56.9	3.5	46.2	3.1	58.6	1.3	68.7	1.2	63.4	1.7	48.2	1.9	33.1	0.9	46.0	2.2	43.5	0.8
C15:O_ai	77.6	2.5	107.5	7.0	96.8	6.8	143.3	1.9	122.7	5.1	103.1	1.6	82.8	1.5	51.6	1.8	76.4	2.5	68.7	4.6
C14:1_c9_(n-5)	160.7	4.9	135.0	9.2	168.4	9.2	235.9	1.2	182.1	8.1	44.8	2.8	26.3	2.0	20.0	0.8	23.1	0.6	36.9	3.2
C15:0	183.5	7.1	205.5	12.5	216.0	11.9	309.4	6.1	242.4	9.1	248.0	4.8	213.5	4.5	132.3	3.2	197.1	7.0	187.3	3.0
C16:0_iso	5.7	0.4	7.9	1.0	7.4	0.5	13.8	0.3	8.5	0.5	14.0	0.6	14.2	1.1	10.6	0.5	14.8	0.0	10.6	0.7
C16:0_ai	41.8	1.7	56.3	6.1	52.5	4.7	71.1	3.9	79.6	4.2	74.2	4.3	62.7	2.6	37.5	0.9	51.2	4.9	51.3	0.4
C15:1_n10_(n-5)	3.8	0.1	3.1	0.3	4.0	0.7	3.4	2.7	4.0	0.1	3.8	0.6	3.3	0.3	1.8	0.3	3.6	0.1	1.7	1.8
C16:0	4582.2	127.6	4084.5	241.7	5401.2	308.5	7441.7	123.0	5385.0	208.7	5803.1	89.9	4343.0	18.8	4085.1	119.3	4222.1	96.3	5692.9	31.7
C17:O_iso	69.1	1.7	96.7	6.2	80.9	4.7	110.2	1.3	103.1	4.9	114.3	3.1	107.5	1.5	72.1	1.9	82.5	1.9	104.3	0.9
C17:O_ai	28.1	1.1	36.2	2.4	35.2	1.8	44.7	5.0	46.7	4.8	71.5	2.1	60.1	0.8	47.9	1.1	50.7	1.2	58.9	0.8
C16:1_t9	73.1	2.1	76.4	4.4	82.7	5.0	144.0	3.0	97.2	4.7	108.0	2.9	95.4	0.6	59.3	0.7	82.7	2.0	82.7	0.4
C16:1_c9_(n-7)	228.0	7.0	162.4	10.8	246.6	14.5	332.2	2.8	245.0	11.7	135.1	4.5	79.2	1.8	88.4	3.5	80.0	2.3	144.7	2.4
C16:1_t10	6.1	0.2	24.4	1.2	7.9	0.4	10.3	0.6	16.5	0.3	6.8	1.1	7.1	1.8	4.7	0.5	5.6	0.3	4.9	0.4
C16:1_t11_+_t12	6.8	1.2	5.1	0.5	7.5	0.1	10.6	1.0	8.9	0.6	4.0	1.2	3.4	2.6	2.8	0.5	2.8	2.1	4.4	0.8
C17:0	113.4	3.2	119.7	7.1	130.8	7.2	194.2	2.8	146.7	6.4	187.7	5.6	164.6	1.8	116.0	3.0	155.2	3.5	131.9	1.2
C18:0_iso	8.9	0.6	10.5	0.8	10.4	0.2	16.6	1.2	14.5	0.4	15.5	0.4	14.7	1.3	8.3	0.7	12.1	0.9	9.4	0.5
C18_0_ai	6.4	0.3	5.7	0.6	6.7	0.4	10.2	0.6	7.9	0.3	11.1	0.4	10.7	1.4	7.2	0.2	8.0	0.2	10.8	0.8
C17:1_c9	5.8	0.3	12.4	0.8	6.1	0.7	9.3	1.4	11.5	0.6	9.3	0.2	7.3	0.1	2.4	0.1	6.7	0.2	6.4	0.3
C17:1_c10_(n-7)	35.5	0.7	30.4	2.2	39.4	2.8	54.0	0.6	42.7	2.6	56.1	1.0	39.5	0.6	32.7	1.1	42.3	0.8	44.2	0.8
C18:0	1527.9	40.6	1869.9	105.9	1669.2	95.8	2282.6	40.7	2210.6	81.6	2552.2	71.5	2946.8	20.9	1953.4	58.5	1771.2	43.9	1994.8	14.3
C18:1_t6	4.1	0.3	3.8	0.4	4.3	0.7	5.6	1.3	4.0	0.4	3.7	2.3	5.9	1.8	2.6	1.4	2.3	1.5	4.1	2.6
C18:1_t7	4.5	0.2	3.7	0.4	5.5	1.0	8.9	1.3	4.1	0.9	5.9	1.2	6.3	1.1	5.0	1.2	3.8	0.4	6.3	0.8

**Table S2** supplementary materials. Fatty acid contend in studied cheese [mg 100g^{-1} of cheese]	S11: Brie de Meaux		S12: Camembert de Normandie		S13: Epoisses de Bourgogne		S14: Petit Brillat Savarin		S15: Pon'tl'evegue		S16: Fromage de chevre au lait cru (Chevre Ronde)		S17: Crottin de Chavignol		S18: Le Fleuret		S19: Picodon Carte Noire		S20: Sainte Maure de Touraine. Hardy Affineur	
	X	SD	X	SD	X	SD	X	SD	X	SD	X	SD	X	SD	X	SD	X	SD	X	SD
C18:1_t8	44.7	1.8	34.7	1.7	45.6	3.1	57.0	3.3	40.8	2.2	49.2	2.5	46.2	0.5	38.4	2.0	27.4	0.5	65.1	1.0
C18:1_t9	45.5	0.7	36.9	2.3	50.2	3.4	60.9	1.9	48.9	0.6	58.9	3.6	57.1	1.1	45.9	1.1	37.4	2.3	74.1	0.1
C18:1_t10	85.6	3.7	0.0	0.0	67.9	5.0	100.3	2.8	486.3	40.2	85.6	1.2	0.0	0.0	62.7	2.7	35.8	1.6	97.7	2.0
C18:1_t11	211.2	4.9	598.8	34.6	339.6	27.4	277.7	1.4	461.1	6.6	276.9	10.5	340.7	2.8	177.8	5.1	132.6	3.4	233.8	1.5
C18:1_t12	64.8	1.9	39.3	2.1	57.7	3.5	79.9	0.8	48.7	2.2	54.5	2.1	75.2	1.3	53.0	1.1	33.0	0.7	70.5	0.5
C18:1_t13+t14	150.7	5.4	119.5	7.3	142.1	9.0	202.4	2.5	132.6	4.2	118.4	2.9	182.7	1.1	109.1	3.7	70.8	2.3	126.0	2.3
C18:1_c9_(n-9c)	2724.6	72.5	2606.6	158.3	3301.4	218.0	3826.0	31.5	3459.3	169.9	4338.6	113.7	4173.8	28.4	3061.6	96.7	2764.5	67.9	4306.1	34.0
C18:1_c10	0.0	0.0	0.0	0.0	0.0	0.0	0.0	0.0	0.0	0.0	4.3	0.3	0.0	0.0	0.0	0.0	0.0	0.0	0.0	0.0
C18:1_c11	85.4	2.8	68.9	4.0	111.0	6.6	126.9	0.2	99.9	4.5	104.1	2.6	89.1	2.1	54.2	2.4	72.1	3.7	112.9	2.1
C18:1_c12	56.6	1.8	19.8	1.3	46.5	2.4	70.6	0.7	34.5	1.4	42.6	0.4	51.8	0.8	39.7	1.4	27.1	0.9	46.3	0.4
C18:1_c13	15.3	0.7	11.6	0.5	16.6	1.3	21.0	0.6	15.8	0.9	12.9	0.4	13.6	0.6	9.6	0.4	8.4	0.2	14.2	0.1
C18:1_c14	69.9	4.0	61.8	3.3	63.7	3.7	91.2	2.7	64.4	2.9	64.4	0.8	98.1	0.6	57.5	2.0	40.8	1.1	60.1	0.9
C18:1_c15	17.3	1.3	15.1	0.6	17.2	1.7	24.6	2.2	20.7	1.5	43.1	1.1	19.1	9.8	32.0	1.2	29.0	6.3	35.1	0.4
C18:1_c16	16.7	1.5	20.9	0.7	19.7	0.9	22.9	0.9	18.1	1.0	0.0	0.0	31.4	0.0	0.0	0.0	9.7	0.5	0.0	0.0
C18:2 _t7_c9	10.7	0.7	16.9	0.6	12.3	0.6	13.6	1.3	15.0	0.4	6.0	3.5	7.5	0.6	5.4	0.4	3.4	1.4	8.7	3.8
C18:2 _c9_t11	6.1	1.2	18.3	0.5	10.0	0.3	7.0	1.3	11.6	1.1	6.0	0.7	5.4	4.0	0.0	0.0	3.2	0.4	0.0	0.0
C18:2_t10_c12	4.1	0.5	3.3	0.4	5.6	0.3	4.7	0.2	0.0	0.0	4.5	0.6	4.4	0.9	2.7	0.2	0.0	0.0	5.0	0.6
C18:2n6t (t9_t12)	44.2	2.0	35.6	1.7	46.3	2.4	51.3	1.7	36.7	1.5	47.0	1.1	68.9	0.5	40.8	1.1	26.1	0.5	61.3	0.5
C18:2_c9_t13	3.4	1.0	2.5	0.2	3.5	0.4	5.6	1.3	4.4	1.1	1.5	0.1	1.2	0.4	0.0	0.0	26.6	0.1	0.6	0.1
C18:2_t9_c13	28.3	0.9	16.9	3.0	30.6	0.2	35.3	1.0	31.4	1.5	20.4	1.8	15.2	1.5	8.9	0.2	10.7	0.2	14.1	0.5
C18:2_t8_c12	19.5	1.8	14.3	1.3	19.2	0.6	23.1	0.9	14.9	0.8	20.6	0.7	32.0	1.5	18.2	0.7	10.1	1.0	27.5	0.3
C18:2_t8_c13	20.4	4.2	15.9	1.1	18.4	0.9	23.4	0.3	17.6	1.4	19.8	1.5	27.8	1.3	16.4	1.6	12.6	0.7	25.6	2.0
C18:2_c9_t12	6.6	0.4	12.4	0.7	8.3	0.6	7.8	0.5	10.2	0.7	6.2	1.2	6.3	0.4	2.8	0.3	3.5	0.4	7.2	0.4
C18:2_t9_c12	26.0	0.5	74.9	4.2	44.1	1.1	38.9	1.3	56.5	2.0	29.3	3.9	63.5	3.1	7.6	5.6	14.7	1.1	10.4	0.8
C18:2n6c (c9_c12)	292.6	6.8	176.1	13.5	351.4	33.7	456.3	0.9	242.2	10.7	565.5	14.7	580.2	2.8	354.4	13.2	348.0	9.3	566.0	4.2
C18:2_t11_c15	0.0	0.0	0.0	0.0	0.0	0.0	0.0	0.0	0.0	0.0	0.0	0.0	0.0	0.0	0.0	0.0	0.0	0.0	0.0	0.0
C18:2_c9_c15	7.6	1.3	6.1	0.9	8.7	0.8	10.6	1.6	6.1	3.6	4.7	3.3	11.3	1.4	0.0	0.0	3.5	0.5	7.4	1.2
C18:3_c6_c9_c12	3.3	0.0	4.9	0.4	3.4	1.2	6.0	1.2	3.7	0.2	0.0	0.0	12.0	0.9	0.0	0.0	0.0	0.0	2.4	0.5
C20:0+C18:3_t9_t12_t15	24.1	1.0	27.1	2.6	26.3	1.6	35.9	0.8	34.3	1.8	59.2	1.8	54.1	0.5	57.0	2.1	53.9	1.8	50.8	1.0
C18:3_t9_t12_c15	0.0	0.0	0.0	0.0	2.8	0.6	0.0	0.0	0.0	0.0	0.0	0.0	0.0	0.0	0.0	0.0	0.0	0.0	1.8	0.4
C18:3_t9_c12_t15	0.0	0.0	0.0	0.0	0.0	0.0	0.0	0.0	0.0	0.0	0.0	0.0	0.0	0.0	0.0	0.0	0.0	0.0	0.0	0.0
C18:3n6 (c6_c9_c12)	4.3	0.9	3.0	0.1	5.6	0.7	9.9	1.9	4.5	0.4	8.5	1.1	5.1	0.4	4.4	0.0	3.9	0.6	5.6	0.9

**Table S2** supplementary materials. Fatty acid contend in studied cheese [mg 100g-1 of cheese]	S11: Brie de Meaux		S12: Camembert de Normandie		S13: Epoisses de Bourgogne		S14: Petit Brillat Savarin		S15: Pon'tl'evegue		S16: Fromage de chevre au lait cru (Chevre Ronde)		S17: Crottin de Chavignol		S18: Le Fleuret		S19: Picodon Carte Noire		S20: Sainte Maure de Touraine. Hardy Affineur	
	X	SD	X	SD	X	SD	X	SD	X	SD	X	SD	X	SD	X	SD	X	SD	X	SD
C18:3_c9_t12_t15+c9_c12_t15	0.0	0.0	0.0	0.0	1.1	0.2	0.0	0.0	3.6	0.7	4.4	0.4	4.1	0.6	0.9	0.2	0.0	0.0	0.0	0.0
C18:3_c9_t12_c15	20.7	0.5	18.1	1.1	3.9	0.7	0.0	0.0	26.5	3.3	4.5	0.7	3.2	0.6	2.2	1.1	3.2	0.6	5.1	1.2
C18:3_t9_c12_c15	14.0	9.5	18.0	1.8	23.2	3.2	29.1	1.0	23.8	0.6	4.6	0.9	2.9	0.1	3.8	0.4	3.5	1.5	6.3	0.9
C20:1	8.3	0.5	6.0	0.6	9.2	1.3	15.1	3.6	8.9	1.0	13.7	2.5	9.0	0.4	9.1	1.7	8.9	0.9	17.2	1.4
C18:3n3 (c9_c12_c15)	75.4	2.4	115.8	10.7	97.3	9.0	113.5	4.9	95.2	3.5	139.2	4.7	375.0	5.7	48.8	2.9	112.3	3.3	71.0	1.3
CLA_c9_t11+t9_c11	104.0	3.6	219.3	13.7	151.4	9.5	131.2	2.8	175.1	7.0	147.2	4.2	142.3	6.0	87.0	1.8	64.0	2.2	161.1	1.5
CLA_c11_t13	5.8	0.3	8.0	1.3	6.4	0.5	10.1	1.5	6.5	3.4	15.0	2.0	14.9	0.8	11.0	0.5	13.8	0.3	9.3	2.6
CLA_t10_c12	0.0	0.0	2.0	0.1	7.0	1.0	11.2	0.9	8.9	1.0	14.1	1.2	1.5	0.1	0.0	0.0	14.5	0.5	11.4	1.6
CLA_c8_c10	3.8	0.6	13.2	1.7	7.6	1.1	6.1	0.5	9.9	0.6	4.6	0.4	13.0	0.4	0.0	0.0	0.6	0.1	0.0	0.0
CLA_c9_c11	0.0	0.0	0.0	0.0	6.2	0.7	1.5	0.2	0.0	0.0	0.0	0.0	1.3	0.3	0.0	0.0	0.0	0.0	0.0	0.0
CLA_c10_c12	0.0	0.0	0.0	0.0	0.0	0.0	0.8	0.2	0.0	0.0	0.0	0.0	0.0	0.0	0.0	0.0	0.0	0.0	0.0	0.0
CLA_c11_c13	4.2	1.0	0.0	0.0	0.0	0.0	0.0	0.0	0.0	0.0	0.0	0.0	0.0	0.0	1.9	0.1	0.0	0.0	0.0	0.0
CLA_t11_t13	3.3	0.1	4.1	0.9	0.0	0.0	0.0	0.0	0.0	0.0	0.0	0.0	7.2	1.9	0.0	0.0	0.0	0.0	0.0	0.0
CLA_t12_t14	7.4	0.7	10.8	0.8	8.5	0.1	9.8	0.6	7.2	0.6	5.6	0.1	8.9	0.5	0.0	0.0	2.4	0.1	3.1	0.5
CLA_t9_t11+t10_t12	3.8	0.8	4.1	0.6	3.4	0.5	5.1	1.6	0.0	0.0	4.3	1.0	2.8	0.3	0.0	0.0	1.7	0.1	3.5	0.3
C18:3_c9_t11_t15	3.1	0.7	7.3	0.4	4.1	0.4	4.6	0.6	6.4	1.6	4.3	1.1	5.3	0.3	0.0	0.0	0.0	0.0	0.0	0.0
C20:2	4.9	0.5	3.3	0.3	5.8	0.2	7.9	1.5	5.6	1.0	5.1	0.4	6.0	0.9	3.6	0.7	4.8	0.3	5.5	0.9
CLA_t8_t10	0.0	0.0	0.0	0.0	0.0	0.0	0.0	0.0	0.0	0.0	0.0	0.0	0.0	0.0	0.0	0.0	0.0	0.0	1.2	0.2
CLA_t7_t9	0.0	0.0	0.0	0.0	0.0	0.0	0.0	0.0	0.0	0.0	0.0	0.0	0.0	0.0	0.0	0.0	1.7	0.1	0.0	0.0
C18:3_c9_t11_c15	7.1	0.4	13.5	0.3	9.2	0.4	12.0	0.9	12.0	0.2	14.4	0.8	34.5	0.9	7.6	0.8	8.8	0.6	7.9	0.2
C22:0	8.9	0.1	11.9	0.9	9.9	1.3	14.3	1.3	14.9	0.5	17.6	2.4	16.8	1.2	18.8	0.7	20.7	1.5	11.5	0.4
C20:3n6	14.6	0.7	10.4	0.6	16.0	1.1	26.8	0.0	16.4	1.1	6.8	0.4	5.2	0.6	5.7	0.1	4.8	0.6	6.7	1.0
C18:4_n3	2.5	0.1	2.8	0.5	2.6	0.1	4.4	0.5	3.8	0.3	0.0	0.0	0.0	0.0	1.6	0.4	1.5	0.2	0.0	0.0
C22:1n9	2.0	0.4	3.2	0.4	2.3	0.8	3.2	0.7	2.8	0.4	3.3	1.5	5.7	0.3	4.1	2.1	2.8	0.5	6.9	1.1
C20:3n3	1.0	0.2	0.9	0.3	3.5	0.6	4.5	0.2	4.5	0.3	3.1	0.9	3.0	0.1	0.0	0.0	2.6	1.8	2.2	0.6
C20:4n6	23.0	1.0	16.6	0.9	28.2	1.3	44.2	0.3	27.9	1.8	46.3	0.7	35.9	2.0	33.3	1.3	29.0	0.1	36.5	1.4
C23:0	4.5	2.2	6.6	5.0	6.8	1.9	9.9	3.3	4.6	4.8	4.2	3.4	4.7	4.8	5.0	2.3	1.7	1.0	1.7	1.4
C22:2	4.8	0.5	12.5	0.7	5.6	0.6	7.6	0.4	9.8	0.5	9.8	0.7	6.8	0.7	1.8	0.1	5.8	0.4	3.7	2.7
C20:5n3 EPA	7.6	0.4	13.8	1.1	8.5	0.3	11.6	1.0	12.2	1.2	13.2	0.6	17.3	0.7	6.6	0.4	10.4	0.4	6.2	0.2
C24:0	8.0	0.5	10.9	0.9	9.2	0.8	13.0	1.1	13.7	1.3	10.9	0.8	8.6	0.8	7.7	0.7	12.4	0.6	5.2	0.1
C24:1	7.3	9.4	6.0	6.5	1.4	1.2	3.7	0.4	3.1	1.0	4.0	0.5	3.0	0.3	1.9	0.4	3.8	0.4	3.0	0.2
C22:6n3 DHA	12.3	9.1	3.8	0.2	1.6	0.7	10.4	15.2	2.4	1.0	9.8	0.6	15.8	1.1	4.2	0.3	8.8	1.2	4.6	1.4

**Table S2** supplementary materials. Fatty acid contend in studied cheese [mg 100g^{-1} of cheese]	S21: Selles sur Cher. Appellation d'origine protégée		S22: English Cheddar Farmhouse		S23: EmmentalerFrancais		S24: RacletteBadozPrest		S25: Tette de Moine		S26: Tomme de SavoieYenn		S27: TommeChevre		S28: Appenzeller extra noir		S29: Beaufort		S30: Comte BadozReserva	
	X	SD	X	SD	X	SD	X	SD	X	SD	X	SD	X	SD	X	SD	X	SD	X	SD
C4:0	603.5	231.4	1070.6	113.3	838.0	95.2	857.6	112.8	1070.2	312.6	855.1	109.4	837.6	105.2	1124.2	142.2	1227.3	169.3	1213.1	312.8
C6:0	567.5	13.0	701.5	25.7	561.4	1.9	606.7	10.8	691.3	24.6	554.5	18.0	642.9	34.7	737.5	11.5	707.1	1.4	747.3	7.6
C8:0	804.4	19.4	519.0	15.2	422.7	4.1	470.4	9.1	523.5	40.0	418.0	14.2	896.1	41.7	552.9	7.5	508.1	3.1	557.3	7.9
C10:0	1982.5	53.2	865.6	19.2	708.1	2.7	823.3	14.8	901.1	127.4	713.2	22.1	2103.7	91.3	893.9	11.8	786.6	6.9	907.8	20.9
C12:0	898.6	22.0	1007.7	15.8	752.9	7.8	887.9	15.2	894.8	15.8	763.4	21.9	883.3	41.8	934.6	19.1	828.8	18.3	952.6	18.4
C13:0_iso	4.6	2.0	10.6	1.4	7.8	1.2	8.8	0.4	11.9	0.9	12.5	0.7	6.5	1.2	12.6	0.4	16.7	0.7	19.6	1.1
C10:1	12.1	1.5	29.9	0.8	21.2	0.1	24.1	0.4	27.1	1.3	20.1	0.2	8.2	0.8	30.0	1.3	21.2	1.0	29.6	2.6
C13:0_ai	21.5	0.6	28.3	1.6	22.6	2.0	23.9	1.3	25.1	2.3	21.6	1.7	15.6	1.5	31.0	4.2	20.1	0.5	29.3	1.9
C14:0_iso	19.7	0.6	27.9	0.6	26.6	0.5	28.7	0.4	43.1	1.0	33.7	0.5	22.6	0.7	32.1	1.2	41.7	0.8	51.9	1.2
C14:0	1810.6	41.1	3011.3	47.4	2425.6	37.0	2732.7	45.6	3116.5	46.7	2566.8	75.7	2014.8	94.7	3108.1	67.5	2867.2	76.9	3305.3	66.0
C15:O_iso	38.1	1.3	72.5	0.8	54.2	1.0	55.3	1.3	89.5	1.2	73.9	2.7	48.8	2.3	72.1	1.7	86.9	1.5	117.8	2.5
C15:O_ai	64.8	2.3	132.3	1.2	97.9	3.0	108.1	1.4	159.5	5.8	139.4	4.3	77.5	4.2	140.5	4.3	156.3	3.1	203.8	4.5
C14:1_c9_(n-5)	32.8	1.3	273.6	4.8	208.2	2.7	220.3	4.1	277.6	15.3	201.8	5.5	27.3	0.8	298.1	6.9	209.1	8.1	269.4	5.2
C15:0	181.0	5.2	312.2	8.0	255.4	7.1	270.9	5.4	342.7	7.7	273.7	8.4	189.5	8.3	321.1	9.8	289.0	12.7	368.4	7.1
C16:0_iso	15.7	0.1	9.6	0.2	8.6	0.6	7.5	0.5	14.5	0.5	13.1	0.3	11.6	1.0	14.9	0.5	27.0	0.4	16.2	1.0
C16:0_ai	55.3	0.5	64.6	8.6	61.9	1.7	74.3	1.4	87.4	5.1	77.1	6.7	57.4	6.9	67.6	12.5	82.4	2.3	96.3	18.7
C15:1_n10_(n-5)	2.0	0.7	8.1	1.3	5.0	1.0	3.4	2.0	4.9	2.2	3.5	1.9	2.3	0.9	6.7	0.5	4.4	0.6	6.1	0.3
C16:0	4115.8	90.5	8309.5	119.5	6457.9	100.4	6945.5	121.8	8849.7	114.9	5871.6	172.1	4715.9	223.4	7553.5	155.8	7064.0	193.4	7495.5	157.8
C17:O_iso	118.7	3.7	115.3	3.3	82.3	1.2	81.3	1.9	133.7	3.1	132.7	3.6	106.1	5.2	151.3	4.6	174.1	5.8	193.1	4.7
C17:O_ai	45.0	1.3	51.8	7.7	36.8	3.7	41.5	4.2	51.1	4.0	52.2	4.6	62.1	2.8	60.2	1.5	75.7	1.4	76.4	1.8
C16:1_t9	88.8	3.3	114.0	5.2	80.7	1.7	94.7	1.0	128.8	2.5	111.4	3.8	90.5	4.3	109.2	2.4	117.6	3.2	145.6	4.0
C16:1_c9_(n-7)	102.7	2.1	445.0	3.7	346.0	5.5	334.9	6.6	419.4	16.4	286.4	7.4	109.9	5.5	386.4	9.9	306.9	9.1	357.2	7.9
C16:1_t10	10.2	0.1	17.7	1.1	15.4	1.0	9.1	0.6	20.8	0.7	17.0	0.5	6.6	0.2	41.8	1.8	34.0	1.5	38.5	1.0
C16:1_t11_+_t12	5.2	4.9	12.1	0.7	9.3	0.5	9.2	1.0	11.0	0.4	8.4	1.1	4.7	1.6	11.2	0.3	9.4	0.9	9.2	0.8
C17:0	145.8	3.7	178.1	3.7	149.9	2.7	160.6	3.4	215.1	3.3	167.3	3.8	160.1	7.7	192.1	4.6	201.2	4.6	219.3	4.3
C18:0_iso	13.8	0.6	15.7	0.8	14.3	1.0	13.3	0.3	22.2	1.0	16.9	0.6	13.6	0.5	20.5	5.9	22.7	1.5	22.2	1.9
C18_0_ai	9.8	1.2	11.0	0.7	8.0	0.1	9.3	0.6	10.7	0.3	7.8	1.0	9.3	0.2	8.7	0.4	11.1	0.6	12.1	0.7
C17:1_c9	4.6	0.7	19.1	0.5	12.9	0.5	7.7	0.3	19.5	0.7	12.9	0.6	8.4	0.4	17.9	1.0	17.2	0.7	25.2	0.3
C17:1_c10_(n-7)	39.6	0.8	62.8	1.7	51.4	0.6	50.2	1.7	70.9	0.2	51.7	1.4	52.3	2.6	63.8	2.0	63.5	2.1	72.7	1.9
C18:0	1641.4	40.6	2547.8	40.7	1903.2	31.7	1928.1	33.9	2540.8	55.0	2362.7	70.6	2483.1	122.3	2626.5	59.5	3422.3	87.7	3044.3	57.7
C18:1_t6	3.7	2.4	6.3	1.1	3.1	2.4	4.7	0.5	4.2	2.2	5.6	0.7	4.0	0.2	4.4	3.5	6.2	0.4	5.7	0.3
C18:1_t7	6.1	0.4	6.6	1.5	3.5	2.2	4.2	0.7	5.6	0.7	6.5	1.3	4.9	0.5	7.2	0.3	6.6	0.3	7.0	1.0

**Table S2** supplementary materials. Fatty acid contend in studied cheese [mg 100g-1 of cheese]	S21: Selles sur Cher. Appellation d'origine protégée		S22: English Cheddar Farmhouse		S23: EmmentalerFrancais		S24: RacletteBadozPrest		S25: Tette de Moine		S26: Tomme de SavoieYenn		S27: TommeChevre		S28: Appenzeller extra noir		S29: Beaufort		S30: Comte BadozReserva	
	X	SD	X	SD	X	SD	X	SD	X	SD	X	SD	X	SD	X	SD	X	SD	X	SD
C18:1_t8	62.1	1.1	57.5	2.4	38.1	0.9	44.5	2.6	58.7	3.9	61.0	1.4	48.8	2.4	68.8	1.7	69.6	2.7	68.0	2.5
C18:1_t9	69.3	2.0	64.9	2.5	45.8	2.3	56.4	1.0	68.4	3.3	64.7	4.5	58.8	2.5	72.0	1.7	74.6	0.7	78.8	3.9
C18:1_t10	138.9	5.8	0.0	0.0	355.8	18.7	65.4	3.0	0.0	0.0	0.0	0.0	76.3	4.4	0.0	0.0	0.0	0.0	0.0	0.0
C18:1_t11	451.1	12.6	455.6	9.5	348.0	7.3	201.7	4.9	508.7	7.7	629.3	18.8	303.2	15.1	993.5	24.7	1104.0	26.9	1113.1	23.5
C18:1_t12	79.8	2.6	74.5	0.3	49.9	1.1	61.4	2.1	57.0	4.1	68.1	1.8	61.6	3.2	75.8	2.7	81.3	1.2	69.1	1.4
C18:1_t13+t14	223.3	5.4	211.3	4.0	128.4	2.3	143.8	2.1	141.4	3.2	166.5	5.3	137.7	6.6	198.9	4.1	231.5	5.8	185.6	4.0
C18:1_c9_(n-9c)	2763.8	79.1	4788.1	73.3	3741.1	58.6	3902.2	77.4	4893.3	151.1	4641.9	157.9	4098.8	207.6	5136.3	121.7	5481.2	140.5	5825.6	134.1
C18:1_c10	111.9	11.0	0.0	0.0	0.0	0.0	0.0	0.0	5.3	0.2	0.0	0.0	0.0	0.0	0.0	0.0	0.0	0.0	139.1	5.2
C18:1_c11	97.6	2.3	126.8	1.6	106.2	2.7	122.7	3.1	121.6	3.8	134.4	4.4	94.6	4.5	123.7	2.2	130.7	3.3	147.4	2.2
C18:1_c12	138.1	3.3	50.1	1.6	39.7	0.6	55.0	1.1	33.2	3.4	46.0	2.0	48.1	1.6	38.6	1.1	43.7	1.0	35.9	0.3
C18:1_c13	17.5	0.7	21.9	0.6	17.4	0.7	15.5	1.3	18.7	0.9	19.2	0.9	12.4	1.0	23.8	0.8	23.5	0.7	21.4	1.1
C18:1_c14	81.6	2.4	95.4	2.1	65.4	2.1	67.6	0.1	67.7	3.1	86.1	2.8	66.8	3.7	100.6	1.3	131.3	1.8	98.7	2.6
C18:1_c15	24.2	1.4	23.1	1.8	17.1	0.9	15.0	6.8	30.7	18.5	21.3	0.5	15.6	9.6	22.7	0.9	30.6	1.4	26.4	1.7
C18:1_c16	57.7	15.2	31.7	1.2	22.7	2.7	1.2	0.1	17.5	0.5	26.5	1.3	20.6	1.6	46.1	12.9	42.4	0.7	36.7	1.5
C18:2 _t7_c9	7.2	1.1	16.7	0.9	12.2	0.2	8.8	0.2	17.8	1.8	17.0	1.3	5.2	1.5	21.5	0.5	14.6	2.3	31.3	1.9
C18:2 _c9_t11	12.7	1.1	13.0	0.7	11.8	0.7	4.8	0.3	11.5	1.5	16.3	2.3	5.1	3.5	29.1	1.2	35.8	2.5	30.3	3.4
C18:2_t10_c12	8.2	0.8	7.9	1.0	5.5	1.2	3.8	0.8	5.3	0.2	5.8	0.5	3.7	0.8	5.6	0.9	5.3	0.2	6.5	0.9
C18:2n6t (t9_t12)	90.2	3.6	63.7	2.0	42.8	1.0	46.3	1.9	44.4	1.1	60.3	2.5	54.0	3.0	73.3	1.6	78.9	0.2	74.2	2.4
C18:2_c9_t13	2.7	0.2	5.9	1.5	4.0	0.8	4.0	0.4	4.9	1.3	3.0	0.2	1.3	0.1	1.6	0.3	2.4	0.5	3.4	1.7
C18:2_t9_c13	8.7	1.7	26.9	1.1	26.5	0.7	41.0	0.7	28.1	2.9	37.1	1.5	14.3	1.1	20.9	2.1	40.5	1.6	33.5	2.7
C18:2_t8_c12	35.4	1.9	28.4	2.6	19.9	0.5	20.9	1.9	19.5	0.8	25.7	0.6	24.0	2.2	28.9	1.4	33.6	0.2	30.5	2.0
C18:2_t8_c13	28.1	3.7	25.4	2.1	16.9	1.1	20.7	0.8	21.7	0.7	25.5	1.3	20.1	1.0	25.5	0.9	32.7	1.8	29.6	1.1
C18:2_c9_t12	11.1	0.8	9.5	2.8	7.4	0.6	14.2	12.6	9.8	0.6	13.6	1.6	6.9	1.3	19.8	1.1	21.2	0.5	23.7	0.8
C18:2_t9_c12	85.4	5.1	60.3	2.0	55.3	0.6	29.9	1.0	56.5	2.5	72.1	2.9	44.6	4.0	130.6	4.4	159.8	3.8	134.1	4.6
C18:2n6c (c9_c12)	557.8	17.0	390.1	2.3	302.5	6.2	345.2	5.8	444.7	35.3	450.5	19.2	530.4	26.0	393.0	7.1	576.3	12.3	403.5	11.7
C18:2_t11_c15	12.8	1.5	0.0	0.0	0.0	0.0	0.7	0.0	0.0	0.0	0.0	0.0	0.0	0.0	0.0	0.0	5.9	10.1	9.9	1.3
C18:2_c9_c15	10.6	0.7	12.9	2.6	10.0	1.1	10.0	1.2	10.9	1.6	7.4	3.0	6.9	5.5	13.1	0.6	10.5	1.8	8.9	4.7
C18:3_c6_c9_c12	21.0	1.6	6.4	0.9	4.9	0.8	4.1	0.3	0.0	0.0	5.4	0.3	6.4	1.5	6.7	0.9	7.9	0.5	20.4	22.2
C20:0+C18:3_t9_t12_t15	32.8	3.4	40.5	0.4	32.5	0.5	31.9	0.3	54.9	3.0	37.8	1.5	52.1	3.5	38.5	0.7	58.6	3.2	33.6	22.1
C18:3_t9_t12_c15	6.6	0.6	0.0	0.0	0.0	0.0	0.0	0.0	0.0	0.0	0.0	0.0	0.0	0.0	0.0	0.0	0.0	0.0	0.0	0.0
C18:3_t9_c12_t15	0.0	0.0	0.0	0.0	0.0	0.0	0.0	0.0	0.0	0.0	0.0	0.0	0.0	0.0	0.0	0.0	0.0	0.0	9.0	0.6
C18:3n6 (c6_c9_c12)	5.1	0.2	6.8	0.9	3.6	1.5	5.8	2.0	7.6	0.4	9.1	0.2	5.4	1.1	5.9	0.8	8.1	0.8	7.7	0.4

**Table S2** supplementary materials. Fatty acid contend in studied cheese [mg $100g^{-1}$ of cheese]	S21: Selles sur Cher. Appellation d'origine protégée		S22: English Cheddar Farmhouse		S23: EmmentalerFrancais		S24: RacletteBadozPrest		S25: Tette de Moine		S26: Tomme de SavoieYenn		S27: TommeChevre		S28: Appenzeller extra noir		S29: Beaufort		S30: Comte BadozReserva	
	X	SD	X	SD	X	SD	X	SD	X	SD	X	SD	X	SD	X	SD	X	SD	X	SD
C18:3_c9_t12_t15+c9_c12_t15	3.8	0.5	0.0	0.0	0.0	0.0	0.0	0.0	2.5	0.3	0.0	0.0	0.0	0.0	5.0	0.8	5.4	1.8	4.7	0.4
C18:3_c9_t12_c15	0.0	0.0	36.4	4.6	4.0	0.9	26.3	1.8	5.5	0.2	4.0	0.4	2.7	0.2	5.4	0.6	5.4	0.6	6.1	0.4
C18:3_t9_c12_c15	13.3	1.0	34.3	0.5	28.9	2.6	22.9	10.1	48.7	4.4	32.0	2.9	0.0	0.0	36.0	2.4	43.6	2.0	34.8	18.9
C20:1	72.8	106.9	12.9	0.6	12.0	1.1	10.3	0.4	17.1	2.3	11.8	0.4	11.8	1.7	11.6	1.7	13.0	1.2	83.9	122.1
C18:3n3 (c9_c12_c15)	193.4	15.5	135.5	5.0	124.0	4.1	87.5	1.0	198.5	8.2	183.0	4.9	159.8	6.5	249.3	8.2	476.9	12.6	247.8	10.5
CLA_c9_t11+t9_c11	203.7	6.9	187.1	4.4	161.1	4.2	119.9	1.9	226.3	2.0	296.9	9.1	148.8	7.4	456.2	10.5	444.4	11.0	559.6	12.0
CLA_c11_t13	9.4	1.0	10.0	1.4	7.5	1.0	7.2	0.2	12.1	0.7	9.2	0.7	12.4	1.4	10.4	1.0	15.4	2.5	12.2	1.4
CLA_t10_c12	10.5	1.0	0.0	0.0	1.3	0.0	0.0	0.0	0.0	0.0	0.0	0.0	12.1	1.1	0.0	0.0	14.3	1.4	29.5	2.6
CLA_c8_c10	6.5	0.9	12.6	1.2	13.8	0.9	5.5	0.4	11.4	0.4	13.7	1.1	5.6	0.8	25.7	0.4	36.7	1.3	26.1	1.2
CLA_c9_c11	0.0	0.0	0.7	0.1	0.0	0.0	0.0	0.0	0.8	0.4	0.0	0.0	0.0	0.0	0.0	0.0	1.6	0.5	1.0	0.1
CLA_c10_c12	0.0	0.0	0.8	0.0	0.0	0.0	0.0	0.0	0.0	0.0	0.0	0.0	0.0	0.0	0.0	0.0	0.0	0.0	0.0	0.0
CLA_c11_c13	10.3	1.4	0.0	0.0	0.0	0.0	0.0	0.0	0.0	0.0	0.0	0.0	0.0	0.0	0.0	0.0	0.0	0.0	0.0	0.0
CLA_t11_t13	9.2	0.9	7.9	1.3	0.0	0.0	0.0	0.0	0.0	0.0	0.0	0.0	0.0	0.0	6.9	0.3	7.9	2.4	9.9	1.1
CLA_t12_t14	6.7	1.8	12.5	1.6	7.2	0.8	6.5	0.6	8.4	1.0	10.2	1.3	4.5	0.3	15.9	2.0	15.6	2.1	15.9	1.4
CLA_t9_t11+t10_t12	4.8	1.1	5.4	0.3	3.9	0.3	2.7	0.2	5.1	0.8	4.2	0.3	0.0	0.0	9.3	2.6	6.2	1.9	6.6	1.1
C18:3_c9_t11_t15	4.8	0.8	7.4	0.1	7.3	0.8	3.6	1.1	5.7	0.3	7.4	0.2	3.3	0.4	14.7	1.4	16.9	4.8	17.5	3.1
C20:2	4.8	0.5	7.4	1.4	6.0	1.1	5.6	0.6	9.0	1.3	7.9	0.5	4.6	0.4	5.6	4.7	12.1	1.1	8.6	2.0
CLA_t8_t10	1.3	0.2	0.0	0.0	0.0	0.0	0.0	0.0	0.0	0.0	0.0	0.0	0.0	0.0	0.0	0.0	0.0	0.0	0.0	0.0
CLA_t7_t9	13.2	1.2	0.0	0.0	0.0	0.0	0.0	0.0	0.0	0.0	0.0	0.0	0.0	0.0	0.0	0.0	0.0	0.0	0.0	0.0
C18:3_c9_t11_c15	12.7	0.5	14.7	0.8	12.9	0.9	9.8	1.0	14.0	0.3	17.4	0.8	15.0	1.1	28.1	0.9	32.9	0.6	31.0	2.0
C22:0	7.7	1.1	14.8	0.4	13.0	0.4	11.8	0.7	23.6	1.4	15.5	1.2	14.8	0.9	16.7	0.0	28.4	1.3	21.6	1.9
C20:3n6	4.8	0.5	20.7	1.0	14.8	0.4	18.6	0.8	19.7	0.9	19.9	0.7	6.0	0.2	18.3	1.3	18.2	0.7	19.7	0.6
C18:4_n3	3.8	0.6	5.1	0.2	3.5	0.3	3.3	0.4	6.6	1.0	4.2	0.3	1.8	0.2	5.2	0.2	7.6	0.3	6.6	0.1
C22:1n9	3.4	0.2	3.7	0.2	3.4	0.7	2.7	0.7	5.1	0.9	4.4	0.3	3.6	0.5	5.1	0.3	8.9	0.4	7.1	0.8
C20:3n3	27.7	2.7	3.5	1.6	2.6	1.1	3.4	0.4	5.5	0.1	25.4	1.0	2.7	0.3	0.0	0.0	0.0	0.0	7.6	0.4
C20:4n6	28.2	1.6	30.2	1.0	23.6	1.6	33.8	1.0	31.7	2.5	23.0	16.2	38.5	1.5	25.6	0.9	30.2	1.5	30.0	1.1
C23:0	3.6	0.3	6.6	4.9	4.4	4.6	1.8	0.9	11.6	7.2	6.9	5.5	6.2	2.7	8.8	4.4	7.9	9.9	13.1	6.8
C22:2	3.2	0.2	14.5	0.5	10.1	0.8	5.7	0.5	15.1	0.6	13.7	3.4	7.7	0.7	14.6	1.0	13.4	0.8	21.6	1.7
C20:5n3 EPA	17.1	0.5	15.6	0.6	13.1	0.3	10.0	0.4	20.0	0.7	14.0	2.1	12.0	0.9	21.7	0.8	23.4	0.5	24.0	0.4
C24:0	5.2	0.6	13.5	0.2	11.3	0.1	10.4	0.5	18.1	1.1	10.0	5.5	8.1	0.3	14.7	0.3	25.4	0.6	18.9	1.2
C24:1	2.4	0.4	3.6	0.3	2.7	1.4	2.3	1.3	5.5	0.5	2.8	0.9	2.9	0.4	3.5	1.3	4.8	0.8	4.1	0.6
C22:6n3 DHA	9.3	0.9	3.3	1.8	1.3	1.0	5.2	4.8	5.3	0.9	3.0	0.2	9.0	0.6	5.0	0.4	5.3	1.0	5.2	0.7

**Table S2** supplementary materials. Fatty acid contend in studied cheese [mg 100g^{-1} of cheese]	S31: Gruyeressereserve		S32: ParmiggianoReggiano		S33: ManchegoForlasa		S34: OssauIraty		S35: Bleud'Auvergne		S36: StiltonColston		S37: Fourmed' Ambert		S38: Roquefort Papillon	
	X	SD	X	SD	X	SD	X	SD	X	SD	X	SD	X	SD	X	SD
C4:0	987.6	110.9	825.9	105.7	901.0	53.6	1072.3	83.3	879.0	69.8	1067.8	63.2	1050.9	238.3	1039.6	151.7
C6:0	675.4	25.5	530.2	7.3	758.3	8.3	938.2	15.9	570.5	14.5	678.6	9.7	624.5	24.4	871.5	33.5
C8:0	505.9	17.0	402.0	6.8	923.5	12.5	1126.3	23.9	424.1	15.9	500.9	7.3	459.0	17.7	1058.2	45.3
C10:0	834.2	29.1	688.6	12.7	2181.5	20.6	2500.1	51.4	703.3	20.9	832.5	12.8	747.2	32.2	2568.6	96.4
C12:0	868.0	21.9	750.7	10.2	1233.4	15.9	1266.2	17.6	738.4	3.6	936.6	10.7	785.6	37.0	1431.9	51.2
C13:0_iso	15.6	2.6	8.7	0.9	6.6	1.6	10.8	0.5	11.8	0.9	9.8	1.0	14.6	1.6	12.0	1.2
C10:1	26.6	0.8	22.9	0.5	16.0	0.2	12.7	0.7	20.3	0.4	27.8	0.5	22.2	1.8	15.7	0.8
C13:0_ai	138.5	19.4	21.5	1.8	24.7	1.8	19.8	2.0	19.9	0.2	26.5	1.8	23.7	2.8	26.3	2.9
C14:0_iso	35.3	1.6	30.5	0.5	24.4	0.5	38.4	1.3	31.2	1.0	24.8	0.4	36.3	1.8	43.7	1.6
C14:0	2957.8	69.0	2450.0	32.2	2785.3	43.2	2886.5	35.8	2419.8	15.1	2887.6	42.0	2753.9	137.4	3290.3	111.9
C15:O_iso	79.5	1.8	54.6	1.3	55.5	2.1	76.3	0.9	72.3	1.0	63.2	1.5	84.8	3.7	89.6	3.4
C15:O_ai	153.9	5.1	117.6	2.2	115.3	3.1	132.1	2.1	136.3	2.1	114.8	0.7	152.0	8.4	138.8	4.1
C14:1_c9_(n-5)	272.8	5.6	242.5	4.9	63.8	4.0	44.9	1.7	189.6	2.9	264.2	1.6	229.1	11.7	55.6	1.4
C15:0	317.0	10.5	291.7	3.4	304.6	7.3	294.0	2.9	270.8	3.3	291.2	2.2	282.6	11.1	331.0	10.9
C16:0_iso	14.8	0.7	18.1	0.4	21.4	1.3	22.7	0.6	12.6	0.7	9.2	0.8	11.3	0.2	26.0	2.1
C16:0_ai	74.4	1.7	72.4	1.4	73.1	1.4	80.9	5.9	70.4	1.3	66.9	3.6	86.9	5.7	82.5	3.9
C15:1_n10_(n-5)	4.4	3.1	4.7	1.7	5.0	0.8	4.3	0.6	4.5	0.9	5.0	0.3	4.6	0.6	4.0	0.8
C16:0	7570.0	169.3	7516.1	109.5	6116.2	106.9	6299.3	75.2	5905.9	42.3	8489.0	155.2	6943.6	360.6	6964.1	250.3
C17:O_iso	137.1	4.6	89.3	1.8	125.3	8.4	127.9	1.4	129.5	1.7	97.6	3.7	139.3	8.6	114.4	4.5
C17:O_ai	63.2	3.2	50.3	1.1	67.9	0.7	75.6	2.0	58.4	4.9	38.1	1.3	57.9	3.1	71.7	2.3
C16:1_t9	117.6	3.2	96.0	1.0	114.3	1.9	109.6	2.3	110.5	1.4	101.0	1.9	122.2	5.8	116.1	3.7
C16:1_c9_(n-7)	384.5	9.8	398.0	7.8	253.0	5.3	188.6	1.5	321.0	2.7	432.0	3.6	392.6	21.0	218.0	7.4
C16:1_t10	31.3	1.3	9.2	1.3	8.1	1.1	14.5	1.4	22.3	0.7	12.2	0.8	24.9	1.0	12.7	0.2
C16:1_t11_+_t12	9.2	0.6	9.6	1.3	6.0	1.2	5.2	0.3	8.9	0.4	11.4	1.2	11.8	0.9	6.3	0.1
C17:0	192.0	4.5	172.2	1.4	195.9	3.9	189.9	2.1	173.1	0.8	169.9	5.2	186.3	11.2	217.9	8.3
C18:0_iso	18.9	0.8	14.7	0.3	20.0	1.6	20.7	2.5	17.9	0.6	14.6	1.0	20.2	1.5	22.7	2.6
C18_0_ai	9.1	1.1	10.4	1.2	14.0	0.4	12.1	0.2	8.3	0.9	10.0	1.0	10.1	0.3	12.0	0.7
C17:1_c9	18.2	0.4	5.0	0.5	4.8	0.7	8.1	0.2	16.8	0.2	16.6	0.8	16.8	0.3	10.2	0.3
C17:1_c10_(n-7)	65.1	1.3	59.0	0.9	73.8	3.1	54.4	0.9	63.3	0.2	54.4	1.0	70.8	3.5	67.4	2.1
C18:0	2511.7	65.2	2000.3	25.5	2334.8	38.1	2891.5	41.3	2333.4	19.5	2303.6	57.2	2666.1	146.2	2127.0	90.0
C18:1_t6	5.5	0.2	6.3	0.6	6.1	0.4	6.9	1.0	3.6	2.6	5.7	0.5	6.0	1.2	2.2	1.2
C18:1_t7	5.5	0.9	5.5	1.0	8.9	1.2	6.8	0.5	6.2	1.5	5.8	0.2	5.1	0.8	5.1	1.3

**Table S2** supplementary materials. Fatty acid contend in studied cheese [mg 100g-1 of cheese]	S31: Gruyeressereserve		S32: ParmiggianoReggiano		S33: ManchegoForlasa		S34: OssauIraty		S35: Bleud'Auvergne		S36: StiltonColston		S37: Fourmed' Ambert		S38: Roquefort Papillon	
	X	SD	X	SD	X	SD	X	SD	X	SD	X	SD	X	SD	X	SD
C18:1_t8	54.4	2.6	58.7	1.1	80.1	1.9	61.7	2.7	44.5	2.2	54.7	2.2	50.7	3.8	29.4	0.8
C18:1_t9	58.5	1.2	57.6	0.3	71.2	2.5	72.2	2.8	56.9	1.3	62.1	1.7	65.4	3.8	50.7	3.7
C18:1_t10	0.0	0.0	89.3	1.3	425.5	7.3	0.0	0.0	0.0	0.0	81.0	2.2	0.0	0.0	42.0	2.7
C18:1_t11	680.7	18.2	182.0	4.3	254.4	6.3	529.2	6.9	624.1	4.8	261.7	9.3	572.9	31.1	282.5	29.6
C18:1_t12	61.1	1.2	62.8	1.1	86.6	2.2	84.9	1.4	51.9	1.2	74.7	1.9	60.0	4.6	41.8	2.7
C18:1_t13+t14	183.3	4.7	153.2	2.0	205.7	3.6	212.4	3.4	143.5	0.5	191.3	4.8	155.8	8.5	107.8	6.2
C18:1_c9_(n-9c)	4663.1	121.6	4583.7	67.8	4387.2	70.5	4361.3	58.9	4669.9	32.7	4293.6	51.2	5600.4	301.4	3848.0	154.8
C18:1_c10	0.0	0.0	0.0	0.0	0.0	0.0	0.0	0.0	0.0	0.0	0.0	0.0	0.0	0.0	7.8	0.4
C18:1_c11	112.6	2.0	149.5	2.8	150.3	2.8	110.3	1.9	124.8	0.8	121.5	2.2	150.2	7.9	94.5	4.8
C18:1_c12	32.4	2.3	60.3	0.7	82.5	1.8	59.2	2.9	28.8	0.8	49.8	1.7	40.4	3.8	35.9	2.1
C18:1_c13	19.4	0.7	34.7	23.8	17.4	0.6	18.2	1.4	19.6	1.2	19.5	0.7	22.8	2.4	13.2	1.7
C18:1_c14	85.1	1.8	63.9	1.0	81.0	1.5	109.7	4.6	76.0	1.3	83.6	1.7	81.7	5.3	63.1	3.8
C18:1_c15	21.9	1.2	37.7	2.5	14.2	1.4	31.3	14.5	22.4	0.6	18.2	8.6	24.6	3.8	50.3	10.8
C18:1_c16	30.6	1.9	0.7	0.1	0.0	0.0	34.5	0.5	24.3	2.0	22.4	2.0	25.4	2.6	20.1	0.4
C18:2 _t7_c9	18.3	0.5	7.3	2.7	17.7	1.1	11.7	3.7	16.3	1.0	11.7	2.7	15.3	4.2	7.3	0.2
C18:2 _c9_t11	17.3	1.1	5.0	0.3	2.9	0.2	13.4	2.4	16.8	0.5	6.2	1.1	14.7	1.3	7.0	0.4
C18:2_t10_c12	4.9	0.4	0.0	0.0	6.2	0.4	5.7	1.4	4.7	0.3	21.5	28.2	5.0	0.4	4.3	0.4
C18:2n6t (t9_t12)	61.6	0.6	46.2	0.2	78.2	1.0	79.0	0.8	52.1	1.1	38.9	28.6	56.8	3.6	47.0	3.5
C18:2_c9_t13	3.6	0.3	5.3	0.8	0.7	0.3	1.8	0.3	4.0	0.8	10.6	10.9	3.5	1.0	0.0	0.0
C18:2_t9_c13	23.6	1.5	31.1	0.7	20.0	2.3	26.7	3.3	29.2	0.7	26.4	0.6	38.8	4.2	21.5	1.2
C18:2_t8_c12	26.2	0.2	21.1	0.6	31.5	2.2	36.0	2.5	22.0	0.6	25.3	1.9	26.5	1.7	23.3	1.9
C18:2_t8_c13	22.3	1.0	20.9	1.4	28.4	0.2	33.6	1.8	21.1	0.8	19.0	10.3	24.3	2.4	20.4	1.9
C18:2_c9_t12	14.3	1.2	6.7	0.8	6.4	1.1	10.6	0.4	13.3	0.8	15.7	14.8	13.6	1.9	6.8	1.7
C18:2_t9_c12	86.0	0.8	15.8	0.9	33.9	4.7	72.4	1.3	71.4	0.8	26.5	17.7	69.7	3.3	46.1	4.1
C18:2n6c (c9_c12)	373.5	7.9	525.1	8.0	725.5	8.6	559.1	9.8	344.4	3.2	423.6	8.8	406.0	19.7	517.6	22.7
C18:2_t11_c15	0.0	0.0	0.0	0.0	0.8	0.1	0.0	0.0	0.0	0.0	0.0	0.0	0.0	0.0	0.0	0.0
C18:2_c9_c15	11.8	0.7	11.3	1.4	3.1	0.2	9.4	2.3	10.1	0.2	11.0	2.0	11.9	1.6	5.3	2.7
C18:3_c6_c9_c12	6.3	1.6	0.0	0.0	0.0	0.0	6.4	1.2	5.3	1.1	6.4	1.5	5.4	0.9	4.2	0.1
C20:0+C18:3_t9_t12_t15	41.2	1.6	36.2	0.7	69.6	0.8	72.7	0.5	39.5	0.9	49.9	3.2	42.0	2.5	82.8	4.4
C18:3_t9_t12_c15	0.0	0.0	0.0	0.0	5.9	1.5	5.8	0.6	7.3	0.6	0.0	0.0	0.0	0.0	0.0	0.0
C18:3_t9_c12_t15	0.0	0.0	0.0	0.0	0.0	0.0	0.0	0.0	0.0	0.0	0.0	0.0	3.4	0.9	0.0	0.0
C18:3n6 (c6_c9_c12)	8.0	1.2	10.2	1.2	18.7	0.6	14.3	1.1	6.7	1.2	8.2	1.5	8.0	1.1	10.6	1.5

**Table S2** supplementary materials. Fatty acid contend in studied cheese [mg $100g^{-1}$ of cheese]	S31: Gruyeressereserve		S32: ParmiggianoReggiano		S33: ManchegoForlasa		S34: OssauIraty		S35: Bleud'Auvergne		S36: StiltonColston		S37: Fourmed' Ambert		S38: Roquefort Papillon	
	X	SD	X	SD	X	SD	X	SD	X	SD	X	SD	X	SD	X	SD
C18:3_c9_t12_t15+c9_c12_t15	0.0	0.0	0.0	0.0	0.0	0.0	0.0	0.0	0.0	0.0	1.6	0.4	1.0	0.1	0.0	0.0
C18:3_c9_t12_c15	5.0	0.4	33.0	2.0	0.0	0.0	0.0	0.0	30.0	1.9	49.8	9.0	35.4	4.1	4.1	0.2
C18:3_t9_c12_c15	36.3	2.9	26.3	10.4	0.0	0.0	0.0	0.0	32.4	0.2	30.0	18.8	38.3	2.9	0.0	0.0
C20:1	10.9	0.6	13.1	0.8	14.3	0.3	11.0	0.6	11.7	0.9	14.3	0.6	14.6	0.7	12.1	1.5
C18:3n3 (c9_c12_c15)	251.6	8.8	125.6	1.6	117.5	1.6	225.4	3.4	178.1	2.7	105.2	1.2	170.9	7.8	269.5	13.5
CLA_c9_t11+t9_c11	302.8	8.2	111.6	2.9	159.3	4.6	226.5	5.2	280.8	2.8	137.4	4.4	262.4	15.4	147.3	7.9
CLA_c11_t13	9.7	0.6	10.5	1.8	23.0	1.0	25.5	3.4	9.1	0.5	10.1	0.6	9.7	1.3	27.7	2.0
CLA_t10_c12	0.0	0.0	0.0	0.0	0.0	0.0	0.0	0.0	0.0	0.0	0.0	0.0	1.6	0.4	29.1	1.4
CLA_c8_c10	14.1	0.8	0.0	0.0	0.0	0.0	11.7	0.6	14.2	0.3	7.7	0.8	13.2	1.8	6.5	0.4
CLA_c9_c11	0.0	0.0	0.0	0.0	0.0	0.0	0.9	0.0	1.3	0.0	0.0	0.0	0.0	0.0	0.0	0.0
CLA_c10_c12	0.0	0.0	0.0	0.0	0.0	0.0	0.0	0.0	0.0	0.0	0.0	0.0	0.0	0.0	0.0	0.0
CLA_c11_c13	0.0	0.0	0.0	0.0	0.0	0.0	0.0	0.0	0.0	0.0	0.0	0.0	0.0	0.0	0.0	0.0
CLA_t11_t13	3.2	0.1	0.0	0.0	0.0	0.0	0.0	0.0	0.0	0.0	0.0	0.0	0.0	0.0	0.0	0.0
CLA_t12_t14	13.7	1.5	6.6	1.1	8.6	3.0	12.9	1.4	9.5	0.3	8.5	0.6	11.0	0.4	6.3	0.6
CLA_t9_t11+t10_t12	6.2	0.9	4.7	0.5	4.4	0.9	7.5	2.3	4.4	0.8	4.9	1.2	4.6	0.8	9.3	1.1
C18:3_c9_t11_t15	10.7	1.3	6.4	0.9	0.0	0.0	8.1	2.2	7.6	0.6	6.7	2.5	8.1	0.4	5.7	1.0
C20:2	8.1	0.2	9.1	0.1	7.7	0.6	10.3	0.6	6.0	0.4	7.2	0.8	6.2	1.1	7.5	1.4
CLA_t8_t10	0.0	0.0	0.0	0.0	0.0	0.0	0.0	0.0	0.0	0.0	0.0	0.0	0.0	0.0	0.0	0.0
CLA_t7_t9	0.0	0.0	0.0	0.0	0.0	0.0	0.0	0.0	0.0	0.0	0.0	0.0	0.0	0.0	0.0	0.0
C18:3_c9_t11_c15	20.7	0.8	6.6	0.8	15.4	1.0	33.2	0.9	18.0	0.9	9.7	0.2	15.8	0.9	21.8	2.1
C22:0	17.6	1.2	15.0	0.5	28.4	1.2	24.6	14.7	17.4	1.0	18.4	0.6	18.1	0.6	40.6	2.8
C20:3n6	19.3	0.6	29.9	0.9	11.6	1.0	9.0	0.3	15.6	1.2	22.2	0.6	19.2	0.6	7.2	0.7
C18:4_n3	4.9	0.7	5.4	1.1	2.9	0.8	5.3	1.2	3.6	2.2	5.4	0.5	4.4	0.3	0.0	0.0
C22:1n9	6.0	0.2	3.5	1.3	4.1	0.5	3.7	0.3	4.5	0.6	8.1	4.7	4.0	0.7	4.9	1.0
C20:3n3	6.5	0.8	3.0	0.4	0.0	0.0	39.3	3.6	4.4	0.4	5.2	1.2	5.3	0.9	5.5	1.3
C20:4n6	28.8	1.2	45.9	1.5	72.1	2.3	38.3	12.1	25.9	1.3	32.8	1.4	33.9	3.2	43.9	3.0
C23:0	10.0	8.3	4.5	4.1	2.0	1.1	2.4	1.4	5.1	3.3	7.7	4.4	9.7	6.5	9.9	12.7
C22:2	13.7	0.5	4.2	0.4	5.3	0.3	16.7	1.6	14.2	1.6	12.9	0.7	13.5	0.8	20.9	1.6
C20:5n3 EPA	22.7	1.2	11.0	1.5	11.6	0.6	18.6	2.3	17.7	0.4	13.6	0.8	16.3	0.7	20.5	1.0
C24:0	17.7	1.6	12.7	0.5	18.1	7.3	24.0	9.7	16.7	1.7	16.6	4.6	16.8	1.3	22.3	2.8
C24:1	2.8	1.3	1.8	0.6	4.2	0.7	5.3	2.4	3.8	0.6	4.4	0.1	3.0	1.7	7.9	0.4
C22:6n3 DHA	4.5	0.5	19.3	13.4	10.5	0.4	14.2	0.9	3.3	0.4	6.2	2.8	3.5	0.9	15.3	0.8

**Table S3** supplementary materials.<br>Group of fatty acid content in studied cheese<br>[mg 100g^{-1} of cheese]	SFA		SCSFA		BCFA		MUFA		PUFA		n-3		n-6		*trans*-MUFA		*trans*-PUFA		CLA	
	X	SD	X	SD	X	SD	X	SD	X	SD	X	SD	X	SD	X	SD	X	SD	X	SD
S1: Camembert l'aromatique	9.78	0.34	2.15	0.07	0.35	0.01	3.03	0.11	0.34	0.00	0.09	0.01	0.23	0.01	0.55	0.02	0.30	0.01	0.15	0.01
S2: Camembert de Caractere. Roi du Chateau	17.29	0.14	3.96	0.08	0.55	0.01	5.93	0.05	0.64	0.01	0.14	0.00	0.47	0.01	0.79	0.02	0.46	0.04	0.20	0.00
S3: BucheFondante	13.20	0.67	4.68	0.24	0.35	0.02	3.78	0.20	0.69	0.04	0.15	0.01	0.52	0.03	0.51	0.03	0.27	0.02	0.14	0.00
S4: Gorgonzola cremoso	10.49	0.15	2.01	0.04	0.29	0.04	3.35	0.04	0.51	0.02	0.10	0.01	0.40	0.01	0.49	0.01	0.31	0.03	0.09	0.00
S5: Gorgonzola intense	15.89	0.30	3.55	0.15	0.42	0.01	5.37	0.10	0.79	0.01	0.12	0.00	0.66	0.01	0.73	0.02	0.43	0.00	0.14	0.00
S6: Cow mountain cheese non smoked	12.81	0.11	2.71	0.05	0.53	0.00	5.14	0.06	0.63	0.00	0.24	0.00	0.37	0.00	0.80	0.01	0.42	0.03	0.25	0.01
S7: Cow mountain cheese smoked	14.23	0.07	3.23	0.13	0.58	0.01	5.43	0.09	0.75	0.03	0.31	0.02	0.42	0.01	0.89	0.02	0.56	0.02	0.28	0.01
S8: Sheep mountain cheese "oscypek"non smoked	14.14	0.71	4.00	0.26	0.72	0.06	4.94	0.40	0.80	0.11	0.37	0.05	0.39	0.06	1.43	0.09	0.78	0.04	0.68	0.05
S9: Sheep mountain cheese "oscypek" smoked	15.48	0.17	4.94	0.23	0.74	0.01	5.05	0.05	0.75	0.02	0.35	0.01	0.36	0.00	1.63	0.03	0.82	0.01	0.78	0.04
S10: L'ami du Chambertin	12.62	0.73	2.85	0.05	0.42	0.03	3.81	0.26	0.51	0.03	0.11	0.01	0.39	0.02	0.66	0.05	0.38	0.02	0.19	0.01
S11: Brie de Meaux	10.54	0.32	2.32	0.10	0.31	0.01	3.45	0.11	0.45	0.01	0.10	0.01	0.35	0.01	0.55	0.01	0.37	0.02	0.13	0.00
S12: Camembert de Normandie	10.83	0.73	2.67	0.24	0.43	0.03	3.18	0.19	0.37	0.03	0.14	0.01	0.22	0.02	0.82	0.05	0.39	0.02	0.26	0.02
S13: Epoisses de Bourgogn	12.52	0.84	2.89	0.31	0.38	0.02	4.07	0.26	0.54	0.05	0.11	0.01	0.41	0.04	0.67	0.02	0.38	0.02	0.19	0.01
S14: Petit Brillat Savarin	16.83	0.57	3.72	0.35	0.53	0.01	4.87	0.04	0.71	0.02	0.14	0.02	0.55	0.00	0.76	0.00	0.46	0.01	0.18	0.00
S15: Pon'tl'evegue	13.37	0.67	3.06	0.28	0.52	0.02	4.23	0.20	0.43	0.02	0.12	0.00	0.30	0.01	1.22	0.04	0.40	0.02	0.21	0.01
S16: Fromage de chevre au lait cru (chevre Ronde)	18.08	0.44	6.65	0.31	0.53	0.01	4.89	0.12	0.81	0.02	0.17	0.00	0.63	0.02	0.65	0.02	0.31	0.01	0.19	0.01
S17: Crottin de Chavignol	14.91	0.11	5.12	0.13	0.45	0.00	4.66	0.02	1.07	0.01	0.41	0.01	0.65	0.00	0.64	0.01	0.46	0.01	0.19	0.00
S18: Le fleuret	11.53	0.42	3.76	0.19	0.30	0.01	3.42	0.11	0.46	0.02	0.06	0.00	0.40	0.01	0.45	0.01	0.23	0.01	0.10	0.00
S19: Picodon Carte Noire	11.92	0.25	3.74	0.10	0.38	0.01	3.13	0.08	0.54	0.01	0.14	0.00	0.39	0.01	0.36	0.01	0.20	0.01	0.10	0.00
S20: Sainte Maure de Touraine. Hardy Affineur	14.16	0.09	4.23	0.05	0.40	0.01	4.85	0.04	0.72	0.01	0.08	0.00	0.62	0.01	0.64	0.01	0.31	0.01	0.19	0.01
S21: Selles sur Cher. Appellation d'origine protégée	12.80	0.28	4.86	0.22	0.41	0.01	3.56	0.19	0.89	0.00	0.25	0.02	0.63	0.02	0.92	0.03	0.58	0.02	0.28	0.01
S22: English Cheddar Farmhouse	18.60	0.16	4.16	0.06	0.54	0.02	6.00	0.08	0.65	0.01	0.16	0.01	0.47	0.00	0.81	0.02	0.56	0.01	0.24	0.01
S23: EmmentalerFrancais	14.54	0.12	3.28	0.08	0.42	0.01	4.67	0.08	0.52	0.01	0.14	0.01	0.36	0.01	0.95	0.03	0.38	0.00	0.19	0.01
S24: RacletteBadozPrest	15.74	0.21	3.65	0.07	0.45	0.01	4.84	0.09	0.54	0.01	0.11	0.00	0.42	0.01	0.55	0.01	0.40	0.02	0.14	0.00
S25: Tette de Moine	19.25	0.55	4.08	0.51	0.65	0.01	6.03	0.13	0.77	0.03	0.24	0.01	0.51	0.04	0.86	0.01	0.44	0.00	0.26	0.00
S26: Tomme de SavoieYenn	14.62	0.50	3.30	0.17	0.58	0.02	5.57	0.18	0.77	0.03	0.23	0.01	0.52	0.03	0.97	0.03	0.50	0.02	0.33	0.01
S27: TommeChevre	15.01	0.69	5.36	0.27	0.43	0.02	4.58	0.23	0.79	0.04	0.19	0.01	0.59	0.03	0.66	0.03	0.34	0.02	0.18	0.01
S28: Appenzeller extra noir	18.12	0.33	4.24	0.16	0.61	0.03	6.31	0.16	0.76	0.02	0.28	0.01	0.46	0.01	1.38	0.04	0.64	0.02	0.52	0.01
S29: Beaufort	18.02	0.26	4.06	0.14	0.71	0.01	6.53	0.16	1.19	0.03	0.51	0.01	0.65	0.01	1.50	0.04	0.77	0.02	0.54	0.01
S30: Comte BadozReserva	18.90	0.47	4.38	0.30	0.84	0.03	7.19	0.17	0.81	0.02	0.29	0.01	0.49	0.02	1.53	0.03	0.70	0.04	0.66	0.02
S31: Gruyere sse reserve	17.51	0.42	3.87	0.11	0.74	0.03	5.76	0.15	0.76	0.02	0.29	0.01	0.45	0.01	1.02	0.03	0.53	0.00	0.35	0.01
S32: ParmiggianoReggiano	15.70	0.11	3.20	0.08	0.49	0.01	5.68	0.09	0.80	0.02	0.16	0.01	0.62	0.01	0.58	0.01	0.39	0.01	0.13	0.00

**Table S3** supplementary materials. Group of fatty acid content in studied cheese [mg 100g^{-1} of cheese]	SFA		SCSFA		BCFA		MUFA		PUFA		n-3		n-6		*trans*-MUFA		*trans*-PUFA		CLA	
	X	SD	X	SD	X	SD	X	SD	X	SD	X	SD	X	SD	X	SD	X	SD	X	SD
S33: Manchego Forlasa	17.85	0.28	6.00	0.08	0.55	0.02	5.17	0.09	0.99	0.01	0.14	0.00	0.83	0.01	1.06	0.02	0.45	0.01	0.20	0.01
S34: OssauIraty	19.59	0.26	6.90	0.12	0.62	0.01	5.06	0.08	0.97	0.02	0.30	0.00	0.64	0.02	0.89	0.01	0.55	0.01	0.29	0.01
S35: Bleu d'Auvergne	14.50	0.13	3.32	0.07	0.57	0.01	5.60	0.03	0.64	0.01	0.21	0.00	0.41	0.01	0.93	0.01	0.49	0.00	0.32	0.00
S36: Stilton Colston	18.25	0.27	4.02	0.04	0.48	0.01	5.44	0.06	0.66	0.02	0.14	0.00	0.50	0.02	0.67	0.02	0.49	0.04	0.17	0.01
S37: Fourmed'Ambert	16.59	1.01	3.67	0.34	0.64	0.03	6.70	0.36	0.70	0.03	0.20	0.01	0.48	0.02	0.92	0.05	0.53	0.04	0.30	0.02
S38: Roquefort Papillon	20.06	0.85	6.97	0.36	0.64	0.03	4.53	0.19	0.93	0.04	0.31	0.02	0.59	0.02	0.59	0.04	0.32	0.02	0.23	0.01

Table S4 n-6/n3 fatty acid ratio in studied cheese

Studied cheeses	n-6/n-3 ratio
S1: Camembert l'aromatique	2.5
S2: Camembert de Caractere. Roi du Chateau	3.3
S3: Buche Fondante	3.4
S4: Gorgonzola cremoso	3.8
S5: Gorgonzola intense	5.3
S6: Cow mountain cheese non smoked	1.6
S7: Cow mountain cheese smoked	1.3
S8: Sheep mountain cheese "oscypek"non smoked	1.1
S9: Sheep mountain cheese "oscypek" smoked	1.0
S10: L'ami du Chambertin	3.5
S11: Brie de Meaux	3.5
S12: Camembert de Normandie	1.6
S13: Epoisses de Bourgogne	3.6
S14: Petit Brillat Savarin	3.8
S15: Pon't l'evegue n	2.5
S16: Fromage de chevre au lait cru (chevre Ronde)	3.8
S17: Crottin de Chavignol	1.6
S18: Le fleuret	6.5
S19: Picodon Carte Noire	2.9
S20: Sainte Maure de Touraine. Hardy Affineur	7.4
S21: Selles sur Cher. Appellation d'origine protégée	2.5
S22: English Cheddar Farmhouse	2.9
S23: EmmentalerFrancais	2.5
S24: Raclette Badoz Prest	3.8
S25: Tette de Moine	2.2
S26: Tomme de Savoie Yenn	2.2
S27: Tomme Chevre	3.2
S28: Appenzeller extra noir	1.6
S29: Beaufort n	1.3
S30: Comte Badoz Reserva	1.7
S31: Gruyere sse reserve	1.5
S32: Parmiggiano Reggiano	3.8
S33: Manchego Forlasa	5.8
S34: OssauIraty	2.1
S35: Bleu d'Auvergne	2.0
S36: Stilton Colston	3.7
S37: Fourme d'Ambert	2.4
S38: Roquefort Papillon	1.9
